# Characteristics of pulmonary multidrug-resistant tuberculosis patients in Tigray Region, Ethiopia: A cross-sectional study

**DOI:** 10.1371/journal.pone.0236362

**Published:** 2020-08-14

**Authors:** Letemichael Negash Welekidan, Eystein Skjerve, Tsehaye Asmelash Dejene, Mengistu Welday Gebremichael, Ola Brynildsrud, Angelika Agdestein, Girum Tadesse Tessema, Tone Tønjum, Solomon Abebe Yimer

**Affiliations:** 1 Department of Para Clinical Sciences, Norwegian University of Life Sciences, Oslo, Norway; 2 Department of Production Animal Medicine, Norwegian University of Life Sciences, Oslo, Norway; 3 Department of Medical Microbiology and Immunology, Division of Biomedical Sciences, College of Health Sciences, Mekelle University, Mekelle, Ethiopia; 4 Department of Midwifery, College of Health Sciences, Mekelle University, Mekelle, Ethiopia; 5 Department of Bacteriology and Immunology, Norwegian Institute of Public Health, Oslo, Norway; 6 Section for Microbiology, Norwegian Veterinary Institute, Oslo, Norway; 7 Department of Microbiology, Unit for Genome Dynamics, University of Oslo, Oslo, Norway; 8 Department of Microbiology, Unit for Genome Dynamics, Oslo University Hospital, Oslo, Norway; The University of Georgia, UNITED STATES

## Abstract

**Background:**

Tuberculosis (TB) is among the top 10 causes of mortality and the first killer among infectious diseases worldwide. One of the factors fuelling the TB epidemic is the global rise of multidrug resistant TB (MDR-TB). The aim of this study was to determine the magnitude and factors associated with MDR-TB in the Tigray Region, Ethiopia.

**Method:**

This study employed a facility-based cross-sectional study design, which was conducted between July 2018 and August 2019. The inclusion criteria for the study participants were GeneXpert-positive who were not under treatment for TB, PTB patients’ ≥15 years of age and who provided written informed consent. A total of 300 participants were enrolled in the study, with a structured questionnaire used to collect data on clinical, sociodemographic and behavioral factors. Sputum samples were collected and processed for acid-fast bacilli staining, culture and drug susceptibility testing. Drug susceptibility testing was performed using a line probe assay. Logistic regression was used to analyze associations between outcome and predictor variables.

**Results:**

The overall proportion of MDR-TB was 16.7% (11.6% and 32.7% for new and previously treated patients, respectively). Of the total MDR-TB isolates, 5.3% were pre-XDR-TB. The proportion of MDR-TB/HIV co-infection was 21.1%. A previous history of TB treatment AOR 3.75; 95% CI (0.7–2.24), cigarette smoking AOR 6.09; CI (1.65–2.50) and patients who had an intermittent fever (AOR = 2.54, 95% CI = 1.21–5.4) were strongly associated with MDR-TB development.

**Conclusions:**

The magnitude of MDR-TB observed among new and previously treated patients is very alarming, which calls for an urgent need for intervention. The high proportion of MDR-TB among newly diagnosed cases indicates ongoing transmission, which suggests the need for enhanced TB control program performance to interrupt transmission. The increased proportion of MDR-TB among previously treated cases indicates a need for better patient management to prevent the evolution of drug resistance. Assessing the TB control program performance gaps and an optimal implementation of the WHO recommended priority actions for the management of drug-resistant TB, is imperative to help reduce the current high MDR-TB burden in the study region.

## Introduction

Tuberculosis (TB) is a chronic infectious disease, which is most commonly caused by *Mycobacterium tuberculosis (*MTB). TB has continued to be a major global public health concern, being one of the top 10 leading causes of death and the top killer among infectious diseases [1]. It is also the leading cause of death among people living with HIV/AIDS and the main cause of antimicrobial resistance-associated death [2]. Globally, there were an estimated 10 million new TB cases and 1.2 million deaths from TB in 2018 [3].

One of the major factors fuelling the TB epidemic is the emergence and spread of drug-resistant (DR) strains of MTB on new and previously treated cases, which creates a threatening and challenging condition for the prevention and control of TB [4]. Out of the total TB incident cases reported in 2018, 484,000 were resistant to rifampicin (RR-TB), and of these, 78% (3.4% new cases and 18% previously treated cases) had multidrug-resistant TB (MDR-TB). A total of 214,000 patients died due to MDR/RR-TB in 2018 [3].

TB is the most common cause of morbidity and mortality in Ethiopia. The country is among the three highest TB, TB/HIV and MDR-TB burden countries with estimates of 165,000 new TB cases and a rate of 151/100,000 population reported in 2018. In the same year, the number of cases of MDR/RR-TB was 1,600, the number of fatalities from TB was 24,000 for HIV-negative people and an additional 2,200 when including people living with HIV/AIDS [3].

Several studies in Ethiopia assessed the magnitude of drug-resistant TB. A review done by Biadglegne *et al*. reported that the occurrence of MDR-TB among TB patients in Ethiopia ranged from 3.3% to 46.3% [5]. Moreover, based on a recent meta-analysis report, the pooled estimate of MDR-TB among new and previously treated cases was 2% (1 to 2%) and 15% (12 to 17%), respectively [6]. Another study reported a MDR-TB prevalence ranging from 0 to 46.3% [7].

Previous studies in the different Regions of Ethiopia have shown variabilities in the magnitude of MDR-TB. The Oromia Region had 33.2% of MDR-TB cases [8], while in Jigjiga 10.2% of those smear-positive were MDR-TB patients [9]. In studies done in the Amhara Region, southwest Ethiopia and Addis Ababa, the magnitude of MDR-TB was reported to be 36.3%, 27.7% and 39.4%, respectively [10–12].

Early case detection and treatment of TB cases is essential to prevent and control drug-resistant TB. In the Tigray Region where this study was conducted, the treatment success rate was 80%, which is lower than the national and WHO target of achieving a 90% treatment success rate among the detected smear-positive cases. The treatment success rate observed in the Tigray Region was also lower than other regions such as the Afar Region in Ethiopia, which recorded a treatment success rate of 89% [13]. The major factor leading to a poor treatment success rate is treatment failure, which is mostly caused by drug-resistant (DR-TB) strains [13, 14].

In the Tigray Region, there is limited to no data on the burden and associated factors of DR-TB. Therefore, this study aimed at assessing the drug susceptibility pattern of TB for the first- and second-line anti-TB drugs and associated factors among TB patients in the Tigray Region, Ethiopia.

## Material and methods

### Study area

**Fig 1:**
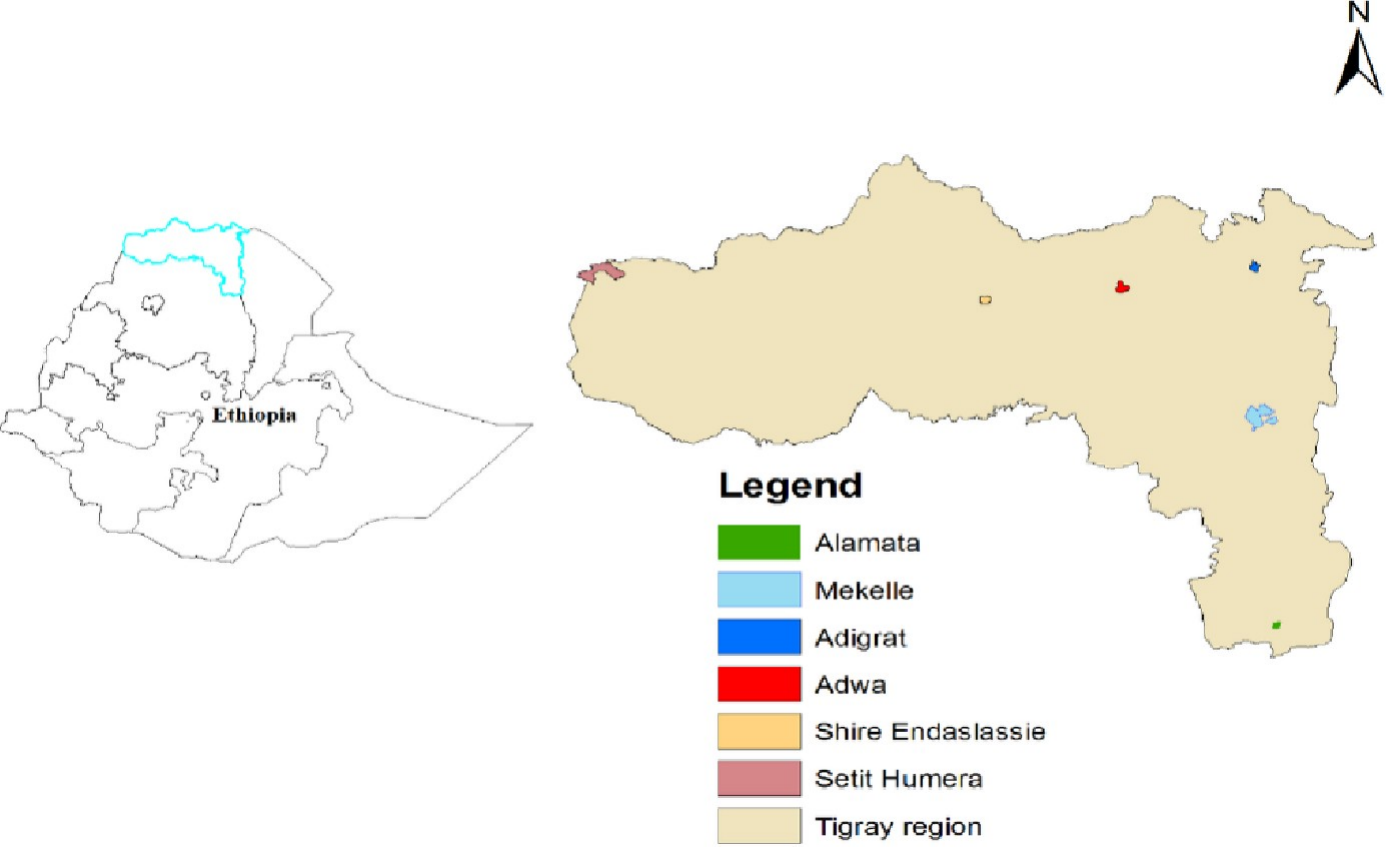
Map of the study area in the Tigray Region, northern Ethiopia, July 2018 to August 2019.

The study was conducted in six hospitals: the Alamata Hospital, Southern Zone; the Mekelle Hospital, Mekelle Special Zone; the Adigrat Hospital, Eastern Zone; the Adwa Hospital, Central Zone; the Shire/Suhul Hospital, Northwestern Zone and the Humera/Kahsay Abera Hospital, Western Zone) of the Tigray Regional State. The region has an estimated total population of 5.13 million [15]. The region is administratively divided into 7 zones (one especial zone, Mekelle), 52 districts and 814 *Kebeles* (lowest administrative unit). The health infrastructure of the region includes 40 hospitals, 223 health centers and 710 health posts serving the population of Tigray and neighboring regions. All the hospitals and health centers are equipped with TB diagnostic facilities (acid-fast bacteria (AFB) smear microscopy) and some hospitals with additional GeneXpert TB diagnostic facility. The Tigray Health Research Institute (THRI) is the only facility providing TB culture and drug susceptibility testing in the Region. The Stop TB (Directly Observed Treatment Short-course (DOTS)) Strategy is being implemented to control TB in the Region. The DOTS-Plus program has been introduced in all health facilities. Currently, there are nine treatment initiation centers and 62 treatment follow-up centers for MDR-TB in the Region. Fig 1 provides the geographical information about the hospitals and zones of the study area.

### Study design, population and inclusion criteria

The study was a hospital-based, cross-sectional study conducted from July 2018 to August 2019. The source population consisted of all presumptive pulmonary TB (PTB) cases in the Tigray Region during the study period. The study population were all presumptive PTB cases who visited the selected health facilities in the region during the study period. The inclusion criteria included PTB patients’ who were not under treatment during the study, ≥15 years of age, who were GeneXpert-positive and could provide written informed consent, were included in the study. Critically ill patients from whom sociodemographic, clinical data and sputum samples could not be obtained, patients <15 years of age and extra-pulmonary TB cases were excluded from the study.

### Sample size determination

The sample size was determined by taking the required minimum number of MDR-TB patients to be enrolled in the study, which was estimated to be between 30–40 cases. This was to establish a sample with sufficient number of MDR-TB isolates to allow a sufficient power to find different varieties of MDR-TB isolates. Based upon an expected level of 10% [9] MDR-TB among TB patients, we aimed at recruiting at least 300 TB patients for the study. This would allow us to sample enough patients for the molecular studies, and also maintain a reasonable level of precision for the prevalence estimates. Without adjusting for clustering, the precision of the estimate would be at 6.7%-14.2% (95% CI, a relative precision of ± 3.3%).

Six government hospitals from the Tigray Region were selected based on the availability of the GeneXpert diagnostic technique as the primary test for presumptive PTB patients from the main zones of the Region without considering other factors (risk of high MDR-TB, high HIV and historically poor TB control program, etc.). The selected hospitals were the only hospitals with the GeneXpert test for the diagnosis of all presumptive PTB patients during the study. These hospitals were also giving diagnosis services for other neighboring health institutes that do not have a GeneXpert test. The standard practice of the hospitals was, first testing of all presumptive PTB patients by GeneXpert test, if the GeneXpert test result is susceptible TB the patient immediately started on 1^st^ line-TB treatment and if the result is RIF-R TB the patient linked to MDR-TB clinic and immediately sputum sample used to be collected for culture before starting MDR-TB treatment.

However, for the purpose of this study, sputum samples were collected for culture and drug susceptibility testing from all consecutive GeneXpert positive PTB patients who fulfilled the inclusion criteria. A consecutive sampling technique was employed to recruit the study sample in all the hospitals until the required sample size was obtained.

### Operational definitions of variables

**New case:** A patient who has never had treatment for TB, or has been on treatment for less than four weeks.

**Previous treatment:** A patient who took TB treatment for one month or more in a previous time.

**Failure after treatment patient**: A patient who has previously been treated for TB, and whose treatment failed at the end of their most recent course of treatment.

**Defaulter (treatment after loss to follow-up):** A patient whose anti-TB treatment was interrupted for two consecutive months or more.

**Relapse:** A patient who has previously been treated for TB, was declared cured or whose treatment was completed at the end of their most recent course of treatment and is now diagnosed with a recurrent episode of TB.

**Mono-drug resistance**: Resistance to only one of the four first-line anti-TB drugs.

**Any drug resistance**: Resistance to one or more first-line anti-TB drugs.

**Multi-drug resistance**: Resistance to at least both isoniazid and rifampicin.

**Rifampicin resistance**: Resistance to rifampicin detected using phenotypic or genotypic methods, with or without resistance to other anti-TB drugs.

**Primary resistance (resistance among new TB cases)**: Resistance in patients who did not have a history of anti-TB treatment.

**Secondary resistance (resistance among previously treated cases**): Resistance in patients previously treated with anti-TB drugs.

**Treatment success rate** refers to the percentage of notified TB patients who were successfully treated.

### Data collection

Socio-demographic and clinical data were collected from patients using a pre-tested structured questionnaire: Age, sex, residence, number of people living in a single room, history of imprisonment, history of TB treatment, contact history with TB patients in the past two years, and TB symptoms like cough, blood in sputum, fever, chest pain, shortness of breath, fatigue, night sweats, loss of appetite and body weight loss. Data on the history of previous medical illnesses and behavioral factors, including alcohol intake, cigarette smoking and Khat chewing habits, were also collected. HIV counselling and testing were performed for TB patients based on the recommendations of the Federal Ministry of Health of Ethiopia (FMOH) testing algorithm [16] at the study hospitals during the study period.

### GeneXpert® MTB/RIF assay

From each patient, a 4 ml sputum sample was collected “on-the-spot” and treated with a sample reagent (SR) containing NaOH and isopropanol according to the recommendation of FMOH [17]. The SR was added in a 2 to 1 ratio of the sputum sample, which was then homogenized and incubated for 15 minutes at room temperature following the manufacturer’s instructions (Cepheid, Sunnyvale, CA, USA) [18]. The treated samples were transferred into the cartridge, and the cartridge was loaded into the GeneXpert instrument. Moreover, the Xpert® MTB/RIF purifies and concentrates MTB from the sputum samples, isolates genomic material from the captured mycobacteria by sonication, and subsequently amplifies the genomic DNA by PCR. The process identifies MTB DNA and rifampicin resistance, thus inducing mutations in the RNA polymerase beta (rpoB) gene in the MTB genome in a real-time format using fluorescent probes called molecular beacons.

### Sputum collection for culturing

A 5–10 ml sputum sample was collected from every GeneXpert MTB/RIF assay-positive participant by laboratory personnel, using a coded and sterile 50 ml falcon tube according to the recommendation of FMOH [17].

All sampled sputa for each participant were properly packed and kept at 4°C for transportation in an ice bag to the Tigray Health Research Institute (THRI), according to the international standards of the WHO recommendation for transport of a biological substance; category B, UN-3373. Specimens arrived within four-five days of collection, and were processed within seven days from the time of first collection.

### Culture and identification

#### Decontamination and sputum processing

Sputum samples were digested using freshly prepared N-acetyl-L-cysteine (NALC) and decontaminated by NaOH (1%). Phosphate buffer (PH 6.8) were added to neutralize NaOH, and dilutes the homogenate to lessen the viscosity and specific gravity. The homogenate was centrifuged at 3000g at 4ºC for 15 minutes [19]. The direct microscopic examination of sputum, and from culture for acid-fast bacteria (AFB) using the standard Ziehl-Neelsen staining, was done at THRI.

#### Sputum culture

The decontaminated supernatant decanted sputum sample was cultured on a Lowenstein-Jensen (LJ) egg medium and on a liquid culture Mycobacterium Growth Indicator Tube; BACTEC MGIT 960 culture (Becton Dickinson Microbiology systems, Sparks, MD, USA), following the standard operational procedures. The tubes for the solid culture were incubated at 37°C in a slant position to ensure an even distribution of inoculums for one week and thereafter at 37°C in air for another seven weeks, and then checked once a week for mycobacterial growth. Cultures were considered negative when no colonies or growth were seen after eight weeks of incubation for a solid culture and six weeks (42 days) for a liquid culture. The growth of mycobacteria were confirmed by its typical colony morphology, acid-fast bacilli (AFB) staining, Capilia antigen test and inoculation onto a blood agar plate to rule out contamination.

### Drug susceptibility testing for first-line and second-line anti-TB drugs using Line Probe Assay (LPA)

Drug-susceptibility testing for first-line anti-TB drugs (isoniazid and rifampicin) using GenoType^®^ MTBDRplus, and second line anti-TB drugs (ofloxacin, levofloxacin, moxifloxacin, amikacin, capromycin, kanamycin and viomycin) using GenoType MTBSL, was carried out by line probe assay genotypic method following the manufacturer’s instruction (GenoType^®^ MTBC; Hain Life Science, Nehren, Germany).

DNA extraction form culture was done using GenoLyse^®^ kit (A and B) in a DNA contaminating free working area. After centrifugation, the supernatant was transferred to a new cryotube. In another room free from contaminating DNA, amplification Mixes A (10 μl) and B (35 μl) were prepared freshly and a total of 45 μl Master Mix was transferred to each polymerase chain reaction (PCR) tubes. The DNA extract (5 μl DNA) was added to respective PCR tubes, and 5 μl of DNA extract from H37Rv quality control strain to the positive control tube and 5 μl of distilled water to negative control tube was added. After amplification, the amplicon was detected with a series of procedures by adding different reagents to the strip. The strips formed color bands after the addition of the final substrate reagent [20].

### Quality assurance and quality control

All laboratory analyses were carried out following standard operating procedures. Both the solid culture and LPA procedures were checked and validated. Reference strains of MTB H37Rv were used as quality control organisms throughout the LPA test. Moreover, both the start and end controls were used during each batch of specimen processing and DNA extraction, as well as no template control being used for LPA reagents.

### Data entry and statistical analysis

Data were double-checked for completeness and cleaned before entry. A data missed by some respondents were traced back to the participants and completed the missed data. A few data which were not very important and missed by most respondents were omitted from the analysis as a whole. If important data were missed by some participants and we could not complete it by tracing back, we included only the respondents in the analysis. The complete and cleaned data were entered using the Epidata 3.1 data entry software. After cleaning and validation, the data were transferred into Stata (Stata SE 15/ SE for Windows, StataCorp, College Station, TX) software for further statistical analysis. The study participants were categorized into two groups: patients with non-MDR-TB and patients with MDR-TB. Descriptive statistics were computed, and frequencies and proportions were presented in tables. Further statistical analyses were performed using a univariable and multivariable logistic regression to identify associations between sociodemographic, behavioral and clinical factors with the main outcome variable (MDR-TB). Results from the logistic regression analysis were presented using Odds Ratios (OR), with 95% Confidence Intervals and p-values. Variables with a p< 0.20 in the univariable analyses were included in the multivariable models. Models were built using a backward selection principle, with a likelihood-ratio (LR) test of p = 0.10 as a cut-of-point for excluding variables. A model fit was assessed using the Hosmer-Lemeshow test and a graphical examination using the sensitivity/specificity and receiver operating characteristic (ROC) curves.

### Ethical considerations

Ethical clearance was obtained from Mekelle University, College of Health Sciences Ethical Review and Research Committee (ERC 1438/2018), Ministry of Science and Higher Education, Ethiopia (SHE/S.M/14.4/708/19) and the Regional Committee for Medical Research Ethics in Eastern Norway (REK Øst) (2018/1118/REK sør-øst A). Written informed consent was secured from all study participants before the commencement of the study.

## Results

**Fig 2:**
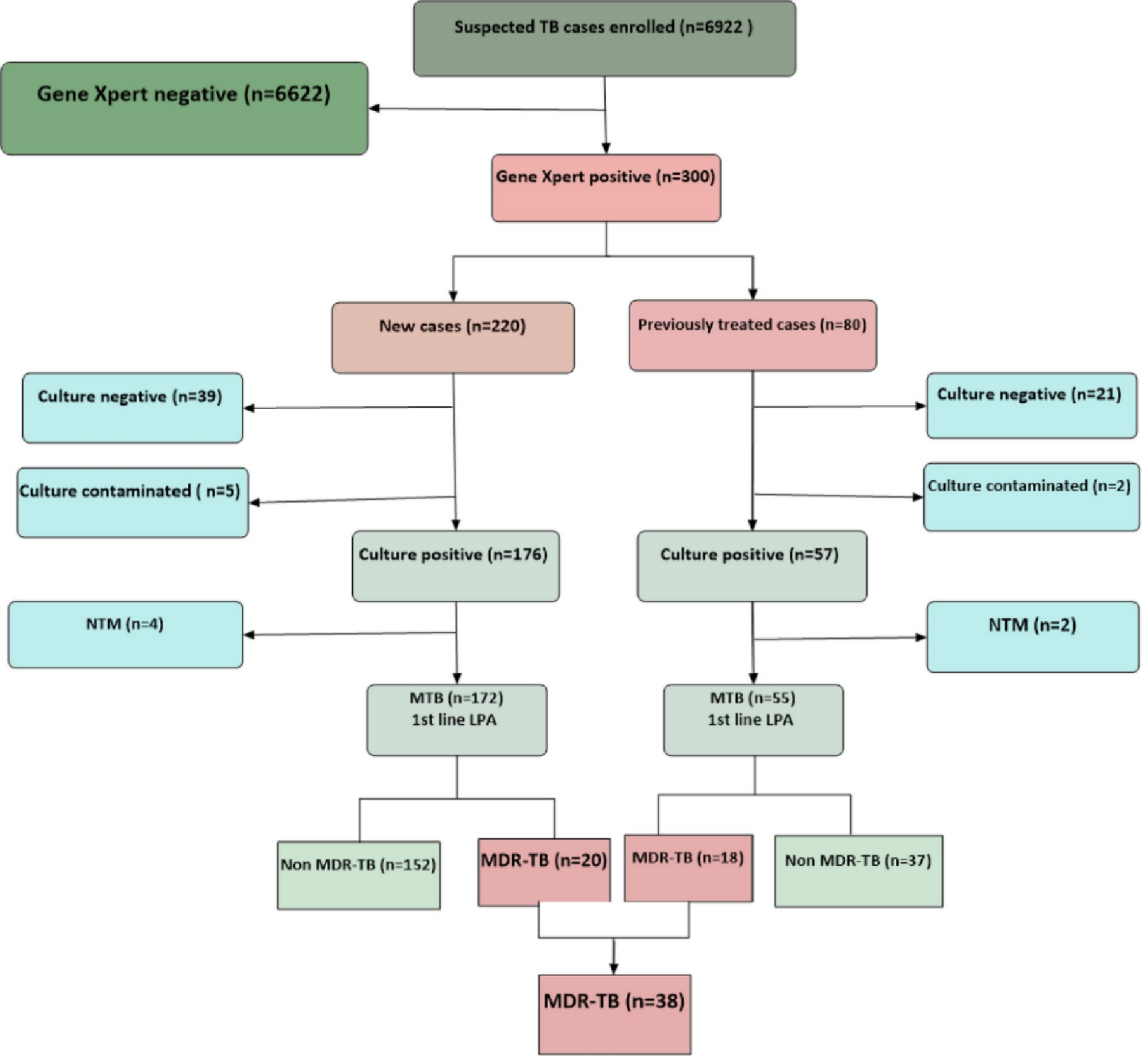
Flow of the procedure followed for all patients recruited in the study in the Tigray Region, northern Ethiopia, July 2018 to August 2019.

A total of 6322 presumptive PTB patients were excluded from the study because of the exclusion criteria stated. Primarily they were GeneXpert negative. In the present study, 300 GeneXpert^**®**^ MTB/RIF assay positive study participants were included. Of these, 227 (75.67%), six (2%), 60 (20%) and seven (2.3%) were MTB culture-positive, non-*Tuberculosis mycobacterium (*NTM), culture-negative and contaminated, respectively. In the rest of this paper, only the 227 study participants with culture-positive tests were used for further analysis. The flow of the study participants’ recruitment process is depicted in Fig 2.

The median age of all 300 participants was 30 years, ranging from 15–85 years. Of these, 196 (65.3%) were males and 104 (34.7%) females. A total of 220 (73.3%) participants were new TB cases, and 80 (26.7%) were previously treated patients. Furthermore, a majority of the participants 112 (37.3%) were in the age group from 25–34 years, 140 (46.7%) were married, 167 (55.7%) were urban dwellers, 98 (32.7%) had only elementary school, 84 (28.0%) were self-employed and 95 (31.7%) had no monthly income.

### Drug resistance patterns of first- and second-line anti-TB drugs

As presented in Table 1, a total of 40 (17.6%) of the 227 isolates were resistant to RIF by GenoType MTBDRplus assay while 42 (18.5%) were resistant to RIF by Xpert^**®**^ MTB/RIF assay. Moreover, six (3.2%) RIF resistant isolates using Xpert^**®**^ MTB/RIF assay were non MDR-TB by the Geno Type MTBDRplus method, and 2/38 (5.3%) RIF susceptible by Xpert^**®**^ MTB/RIF assay were MDR-TB using the Geno Type MTBDRplus method.

**Table 1:** Rifampicin resistance patterns of isolates based on MTB status category of study participants in the Tigray Region, northern Ethiopia, July 2018 to August 2019.

Rifampicin resistance pattern				
	Genotype MTBDR plus			
	Non MDR-TB (n = 189)			MDR-TB (n = 38)
		Susceptible, F (%)	Resistant (%)	Resistant (%)
GeneXpert	Susceptible	183 (96.8)	0	2 (5.3)
	Resistant	4 (2.1)	2 (1.1)	36 (94.7)

Table 2 shows the pattern of resistance among 227 MTB isolates. The dominant isolates 189 (83.3%) were susceptible to INH and/or RIF, two (0.9%) had a mono resistance to RIF, three (1.3%) a mono resistance to INH, 43 (18.9%) a resistance to INH and/or RIF and 41 (18.1%) an overall resistance to INH. The proportion of MDR-TB among new and previously treated patients was 20 (11.6%) and 18 (32.7%), respectively, and the overall MDR-TB was 38 (16.7%). In this study, two (5.3%) of the MDR-TB isolates were pre-XDR-TB, which were resistant to fluoroquinolones (FQs), one of the second-line anti TB drugs. However, for the rest of second-line anti-TB drugs, the MDR-TB isolates were susceptible.

**Table 2:** Drug resistance patterns of isolates to first- and second-line anti-TB drugs with TB patient category of study participants in the Tigray Region, northern Ethiopia, July 2018 to August 2019.

First-line resistance pattern	New cases (n = 172), F (%)		Previously treated cases (n = 55), F (%)	Total cases (n = 227), F (%)
Any-S	152 (88.4)		37 (67.3)	189 (83.3)
RIF-R	21 (12.2)		19 (34.5)	40 (17.6)
RIF mono- R	1 (0.6)		1 (1.8)	2 (0.9)
RIF-S	151 (87.8)		36 (65.5)	187 (82.4)
INH-R	23 (13.4)		18 (32.7)	41 (18.1)
INH mono-R	3 (1.7)		0 (0)	3 (1.3)
INH-S	149 (86.6)		37 (67.3)	186 (81.9)
Any-R	24 (14)		19 (34.5)	43 (18.9)
MDR	20 (11.6)		18 (32.7)	38 (16.7)
**Second-line resistance pattern (38)**	**New cases (20), F (%)**		**Previously treated cases (18), F (%)**	**Total cases (38), F (%)**
FLQ	R	0	2 (11.1)	2 (5.3)
	S	20 (100)	16 (88.9)	36 (94.7)
AMK, CAP, KAN	R	0	0	0
	S	20 (100)	18 (100)	38 (100)
KAN, CAP, VIO	R	0	0	0
	S	20 (100)	18 (100)	38 (100)
KAN, AMK, CAP, VIO	R	0	0	0
	S	20 (100)	18 (100)	38 (100)
Low-level KAN	R	0	0	0
	S	20 (100)	18 (100)	38 (100)

R = resistant, S = susceptible, FLQ = Fluoroquinolones (Ofloxacin, levofloxacin, Moxifloxacin), AMK = Amikacin, KAN = kanamycin, CAP = Capromycin, VIO = Viomycin.

**Fig 3:**
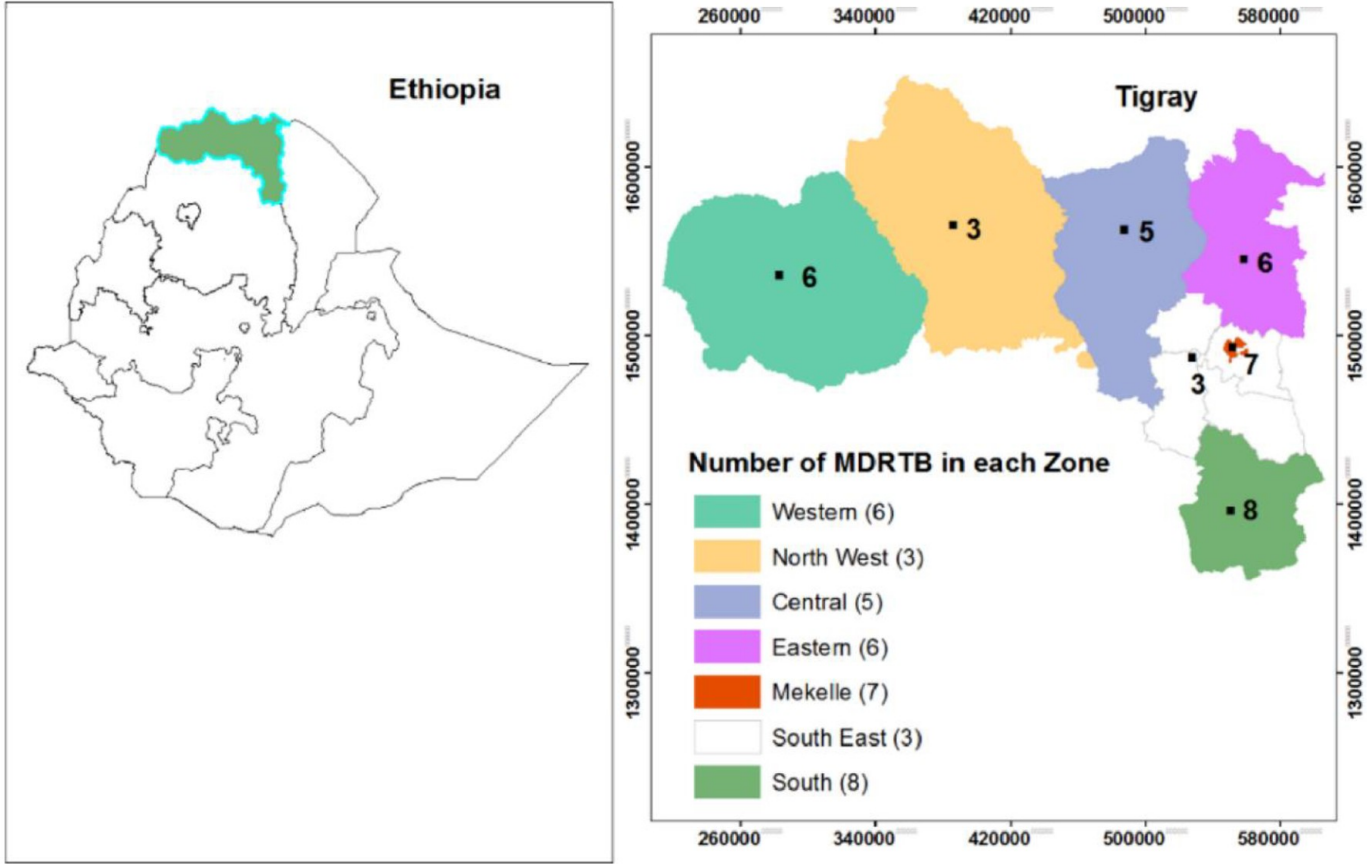
Zonal distribution of 38 MDR-TB among TB patients (N = 227) in the Tigray Region, northern Ethiopia, July 2018 to August 2019.

The proportion of overall TB/HIV, non-MDR-TB/HIV and MDR-TB/HIV co-infection among the 227 TB patients was 41 (33%), 33 (17.5%) and eight (21.1%), respectively. The Zonal distribution showed that MDR-TB was highest in the Southern Zone (21.1%), followed by the Mekelle Special Zone (18.4%) (Fig 3).

### Factors associated with MDR-TB

Patients’ sociodemographic parameters, risk behaviors, MDR-TB/HIV co-infection status, contact history and clinical presentations were compared between non-MDR and MDR-TB cases using univariable and multi-variable logistic regression analysis (Tables 3–5).

**Table 3:** Sociodemographic and behavior factors associated with MDR-TB among the study participants in the Tigray region, northern Ethiopia, July 2018 to August 2019.

Variables	Non-MDR (189), F (%)	MDR (38), F (%)	COR (95% Cl)	P-value	AOR (95% Cl)	P-value
**Sex**						
Male	122 (64.6%)	22 (57.9)	1		-	-
Female	67 (35.5)	16 (42.1)	1.32 (0.65–2.69)	0.438		
**Age in years**						
15–24	38 (20.1)	9 (23.7)	1			
25–34	76 (40.2)	14 (36.8)	0.78 (0.3–1.96)	0.594	**-**	**-**
35–44	36 (19.1)	10 (26.3)	1.17 (0.43–3.22)	0.757		
45–54	20 (10.6)	2 (5.3)	0.42 (0.08–2.14)	0.298		
≥55	19 (10.1)	3 (7.9)	0.67 (0.16–2.75)	0.575		
**Residence**						
Urban	103 (54.5)	24 (63.2)	1			
Rural	86 (45.5)	14 (36.8)	0.7 (0.34–1.43)	0.328	**-**	**-**
**Marital status**						
Single	90 (47.6)	15 (39.5)	1			
Married	86 (45.5)	17 (44.7)	1.18 (0.56–2.52)	0.658		
Divorced	4 (2.1)	2 (5.3)	3 (0.50–17.85)	0.227	**-**	**-**
Widowed	9 (4.8)	4 (10.5)	2.67 (0.73–9.77)	0.139		
**Pregnant (48)**						
No	8 (80)	37 (97.4)	1			
Yes	2 (20)	1 (2.6)	2.53 (0.22–28.6)	0.454	**-**	**-**
**Lactating (48)**						
No	9 (90)	37 (97.4)	1			
Yes	1 (10)	1 (2.6)	5.08 (0.31–3.07)	0.254	**-**	**-**
**Family size**						
≤3	109 (57.7)	19 (50)	1			
≥4	80 (42.3)	19 (50)	1.36 (0.68–2.74)	0.385	**-**	**-**
**Education**						
Illiterate	60 (31.8)	10 (26.3)	1			
1–8 grades	58 (30.7)	16 (42.1)	1.66 (0.69–3.95)	0.256	**-**	**-**
9–12 grades	54 (28.6)	8 (21.1)	0.89 (0.33–2.42)	0.817		
Diploma and above	17 (9)	4 (10.5)	1.41 (0.39–5.07)	0.597		
**Occupation**						
Housewife	20 (10.6)	5 (13.2)	1			
Farmer	46 (24.3)	10 (26.3)	0.87 (0.26 2.87)	0.819	**-**	**-**
Self-employed	58 (30.7)	8 (21.1)	0.55 (0.16–1.88)	0.342		
Government employee	10 (5.3)	2 (5.3)	0.8 (0.13–4.87)	0.809		
Student	13 (6.9)	4 (10.5)	1.23 (0.28–5.45)	0.785		
No work	42 (22.2)	9 (23.7)	0.86 (0.25–2.89)	0.804		
**Monthly income (Birr)**						
<500	41 (21.7)	11 (29)	1			
500–2000	48 (25.4)	10 (26.3)	0.78 (0.3–2.01)	0.603		
˃2000	39 (20.6)	4 (10.5)	0.38 (0.11–1.30)	0.124	-	-
No means of income	61 (32.3)	13 (34.2)	0.79 (0.32–1.94)	0.614		
**Smoking**						
No	176 (93.1)	32 (84.2)	1		1	
Yes	13 (6.9)	6 (15.8)	2.54 (0.9–7.17)	0.079	6.09 (1.7–22.5)	**0.007**
**Alcohol intake**						
No	111 (58.7)	28 (73.7)	1		1	
Yes	78 (41.3)	10 (26.3)	0.51 (0.23–1.11)	0.088	0.28 (0.1–0.77)	**0.014**
**Khat chewing**						
No	173 (91.5)	33 (86.8)	1			
Yes	16 (8.5)	5 (13.2)	1.64 (0.56–4.78)	0.366	**-**	**-**

**Table 4:** Associations of TB/HIV co-infection and contact history with MDR-TB among study participants in the Tigray region, northern Ethiopia, July 2018 to August 2019.

Variables	Non-MDR-TB (%) (n = 189)	MDR-TB (%) (n = 38)	OR (95% Cl)	P-value
**TB patient history in family**				
No	144 (76.2)	26 (68.4)	1	
Yes	45 (23.8)	12 (31.6)	1.48 (0.69–3.16)	0.316
**History of TB close contact**				
No	149 (78.8)	25 (65.8)	1	
Yes	40 (21.2)	13 (34.2)	1.94 (0.91–4.12)	0.086
**History of MDR-TB contact**				
No	176 (93.1)	33 (86.8)	1	
Yes	13 (6.9)	5 (13.2)	2.05 (0.69–6.14)	0.199
**Diabetes mellitus status**				
No	183 (96.8)	36 (94.7)	1	
Yes	6 (3.2)	2 (5.3)	1.69 (0.33–8.73)	0.528
**HIV status**				
Negative	156 (82.5)	30 (79)	1	
Positive	33 (17.5)	8 (21)	1.26 (0.53–3)	0.600
**History of prison**				
No	163 (86.2)	34 (89.5)	1	
Yes	26 (13.8)	4 (10.5)	0.74 (0.24–2.25)	0.593

**Table 5:** Associations of clinical presentations with MDR-TB among study participants in the Tigray Region, northern Ethiopia, July 2018 to August 2019.

Variables	Non-MDR-TB (189), F (%)	MDR-TB (%) (38), F (%)	OR (95% Cl)	P-value	AOR (95% Cl)	P-value
**TB history**						
No	152 (80.4)	20 (52.6)	1		1	
Yes	37 (19.6)	18 (47.4)	3.7 (1.78–7.68)	**0.001**	4.26 (1.99–9.14)	**<0.001**
**Duration of illness in days (198)**						
≤60	104 (62.6)	10 (31.3)	1			
>60	62 (37.4)	22 (68.7)	3.69 (1.64–8.30)	**0.002**	**-**	**-**
**Body mass index**						
>18.5	53 (28)	8 (21)	1			
≤18.5	136 (72)	30 (79)	0.68 (0.29–1.59)	0.377	**-**	**-**
**Weight loss**						
No	18 (9.5)	6 (15.8)	1			-
Yes	171 (90.5)	32 (84.2)	0.56 (0.21–1.52)	0.257	-	
**Chest pain**						
No	57 (30.2)	7 (18.4)	1			-
Yes	132 (69.8)	31 (81.6)	1.91 (0.8–4.6)	0.147	-	
**Coughing for > 2 weeks**						
No	27 (14.3)	6 (15.8)	1			
Yes	162 (85.7)	32 (84.2)	0.89 (0.34–2.33)	0.810	-	-
**Shortness of breath**						
No	92 (48.7)	19 (50)	1			
Yes	97 (51.3)	19 (50)	0.95 (0.47–1.90)	0.882	-	-
**Hemoptysis**						
No	172 (91)	33 (86.8)	1			
Yes	17 (9)	5 (13.2)	1.53 (0.53–4.44)	0.431	-	**-**
**Intermittent fever**						
No	119 (63)	17 (44.7)	1		1	
Yes	70 (37)	21 (55.3)	2.1 (1.04–4.25)	**0.039**	2.54 (1.21–5.4)	**0.014**
**Night sweats**						
No	44 (23.3)	9 (23.7)	1			
Yes	145 (76.7)	29 (76.3)	0.98 (0.43–2.22)	0.957	-	-
**Appetite loss**						
No	68 (36)	9 (23.7)	1			
Yes	121 (64)	29 (76.3)	1.81 (0.81–4.05)	0.148	-	-
**Fatigue and malaise**						
No	142 (75.1)	30 (79)	1			
Yes	47 (24.9)	8 (21)	0.81 (0.35–1.88)	0.617	-	-

A history of previous TB treatment showed a strong association (AOR = 4.26, 95% CI = 1.99–9.14) with MDR-TB development, and patients with cigarette smoking habits (AOR = 6.09, 95% CI = 1.65–22.50) were more likely to develop MDR-TB compared to those who did not smoke. Patients who had an intermittent fever (AOR = 2.54, 95% CI = 1.21–5.4) were two times more likely to develop MDR-TB compared to those who did not experience a fever. Surprisingly, patients who used to consume alcohol (AOR = 0.28, 95% CI = 0.10–0.77) were less likely to develop MDR-TB compared to those who did not take alcohol.

No sociodemographic characteristics and co-infections status were associated with MDR-TB development. This included an absence of association between MDR-TB and HIV infection. In the univariable analysis, patients with a duration of symptoms > 60 days were more likely to develop MDR-TB, but this variable did not show a significant association in the multivariable model.

## Discussion

This is the first and largest study attempting to assess the magnitude and associated factors of MDR-TB in the Tigray Region of Ethiopia. A periodic assessment of the prevalence and associated factors of drug resistance in high TB burden countries is essential to identify early- and address the challenges of drug-resistant TB transmission. This helps to enhance the TB control program performance and achieve the End TB Strategy goals.

The overall proportion of MDR-TB observed in our study was 16.2% (11.6% among new cases and 32.7% among previously treated cases). The WHO data indicates that 6–10% of new TB cases and 13–60% of cases in previously treated patients are MDR [21]. The overall proportion of MDR-TB observed in our study was higher compared to previous studies conducted in Addis Ababa, Ethiopia [22, 23], northwest Ethiopia [24, 25], Jigjiga in the Somali Region [9] and the Oromia Region [26], which showed an overall prevalence of MDR-TB of 11.5%, 5.6%, 10.2%, 1.8%, 1.8% and 4.7%, respectively. The finding was also higher than the study done in China [27], Kenya [28], Tanzania [29], India [30], Vietnam [31], Lima Peru [32], Dalian China [33] and northeastern China [34], where the overall MDR-TB prevalence was estimated at 11.3%, 4.8%, 6.3%, 5.6%, 6.9%, 6.6%, 10.1%, 8.7%, respectively. Conversely, the overall MDR-TB proportion observed in our study was lower than former study findings, which showed a 39.4%, 27.2%, 39.2% and 26% overall MDR-TB prevalence reported from Addis Ababa, Ethiopia [12], southwest Ethiopia [10], Uganda [35] and Taiwan [36], respectively. The increased proportion of MDR-TB observed in our study is very alarming to the TB/MDR-TB control program in the study area, as well as for the country at large.

Increased MDR-TB among new TB cases is an indicator of the ongoing transmission of drug-resistant TB. The proportion of new MDR-TB (11.6%) cases observed in our study is higher than many other studies conducted in Ethiopia, such as eastern Ethiopia (1.1%) [37], northwest Ethiopia (2.3%) [24], Jigjiga, in the Somali Region (4.5%) [9] and the Amhara Region (1.0%) [38]. The finding is also higher than studies conducted in other countries: Tanzania (4.3%) [29], India (2.9%) [30], Vietnam (4.2%) [31], Dalian China (5.8%) [33] and northeastern China (4.2%) [34]. The high proportion of MDR-TB among newly diagnosed TB patients observed in this study is of high concern, which requires an urgent intervention to improve the quality of TB control to interrupt the transmission of drug-resistant TB [39].

The proportion of MDR-TB (32.7%) among previously treated cases in our study was more or less similar with a study report from southwest Ethiopia [40], which showed an MDR prevalence of 31.4%. However, our report was lower than that reported from the St. Peter’s TB Specialized Hospital, Addis Ababa, Ethiopia, which showed an MDR-TB prevalence of 58% [41]. This may not be surprising as the St-Peter’s Specialized Hospital in Addis Ababa is the national referral hospital for specialized TB care in the country. Conversely, our finding was higher than several other previous study results reported from Jigjiga, the Somali Region, Ethiopia [9], Dalian China [33], northeastern China [34] and Vietnam [31], which showed an MDR prevalence of 22.6%, 17.7%, 27.6% and 23.1%, respectively. The increased MDR-TB proportion among previously treated cases in our study indicates a need for better patient management to help prevent the evolution of resistance in the study area.

Five percent of the cases in our study had pre-XDR-TB or FQs-resistant MDR-TB, which is higher than a recent study conducted in Ethiopia (3.4%) [42] and China (0.7%) [34]. Studies in other countries showed a very high proportion of pre XDR-TB compared to our study result: Morocco (22.2%) [43], China (14.5%) [27], Vietnam (17.9%) [31], India (27.6%) [44], western India (22%) [45] and Pakistan (38.7%) [46]. Some of the factors associated with the high pre-XDR prevalence in those countries were related to a widespread use of FQs without prescriptions for the treatment of new TB cases and other undiagnosed respiratory infections in the private sectors [31, 45], with most of the study participants being treatment failures and chronic cases [45]. Overall, the proportion of pre-XDR-TB observed in our study is of great concern, thereby indicating the possibility of an increasing resistance to second-line anti-TB drugs in the region.

Previous studies indicated that several factors such as a previous history of TB treatment (failures, defaulters and relapses), contact with MDR-TB patients and water pipe smoking [23, 46, 47] are all associated with the development of MDR-TB. In our study, patients with a previous history of TB treatment were more likely to develop MDR-TB, with similar findings reported from other studies in Ethiopia and several other countries [6, 36, 40, 48–52]. This association might be related to unsatisfactory/noncompliance by patients or clinicians to anti-TB treatment, a lack of supervision of treatment or poor quality of the DOTS program, improper drug regimens and an inadequate or irregular drug supply, which may potentiate genetic mutations in the bacteria, and can result in acquired drug resistance [48, 53–55].

Cigarette smoking was associated with MDR-TB. This is similar to other study findings [23, 25, 35]. Cigarette smoking directly causes ciliary dysfunction; this diminishes the immunity of individuals, which makes them prone to primary MDR-TB [56, 57].

A univariable analysis showed that patients who experienced TB symptoms for a duration of more than 60 days were more likely to develop MDR-TB compared to their counterparts. This finding is in line with studies and reports from several other countries [40, 48, 58]. One of the mechanisms of MTB drug resistance arises from spontaneous point mutations. Over time, these mutations accumulate, and acquired drug resistance may occur if diagnosis and treatment is not initiated early [59]. A delay in treatment can also result in an overgrowth of MTB (a high grade of sputum positivity), which causes a delayed sputum conversion during treatment, and in turn is associated with an acquired MDR-TB [58, 60, 61].

The current proportion of MDR-TB/HIV co-infection was 21.1%, which is lower than the findings in previous studies in northwest Ethiopia (28.6%) [24], Addis Ababa, Ethiopia (79.8%) [12] and southwestern Ethiopia (43.5%) [40]. Nevertheless, this is higher than the results reported from Mali (10.5%) [47], Thailand (4.8%) [58] and India (5.6%) [30]. HIV infection is the strongest risk factor for the development of active TB regardless of the type of MTB, whether drug-susceptible or primary drug-resistant, as it suppresses the immune system of the individuals [62]. In our study, HIV co-infection was not associated with the development of MDR-TB, which is similar to other findings [40, 47, 58], though some studies reported its association with MDR-TB [37, 38, 49]. A review by Wells *et al*. reported that there was no association between MDR-TB and HIV in several studies in Africa, Russia, Vietnam, India and other multi-country studies. However, this review reported that there was an association in a study done in the United States and in Ethiopia, but in this review the association was only with primary MDR-TB [62]. This review in particular reported that a specific genotype family of drug-resistant strains of MTB might play a role in transmission, especially among people living with HIV infection. The Beijing genotype family, which includes the “W” strain of MTB implicated in many MDR-TB outbreaks in the United States, is more virulent and is associated with an anti-TB drug resistance in specific geographic settings [62].

We were surprised to observe the association between the consumption of alcohol and a lower chance of acquiring MDR-TB. As far as our literature review is concerned, there is no study that supports our finding. Our hypothesis is that this may have happened by chance. Further study is warranted to understand the link between alcohol intake and drug resistance.

In the current study, none of the sociodemographic factors were statistically associated with MDR-TB development, which is similar with other study reports [24, 58, 63]. A recent similar study in the Tigray Region reported that age was marginally associated with MDR-TB [64]. Yet, this result could not be reproduced in our study. Sociodemographic variables such as educational status [65], age [49] and residence [26] were associated with MDR-TB in studies conducted in the various other regions of Ethiopia.

Ethiopia has initiated an innovative community health program (health extension program) in all its regional states, including Tigray, over the last 15 years. This initiative can play a big role in increasing early case detection and an improved treatment success rate by improving service access to TB patients [66, 67]. Regardless of the priceless role of health extension workers in TB/MDR-TB prevention and the control program, a study conducted in the Tigray Regional State reported that only one-fourth (25%) of the health posts were working efficiently [68]. This may contribute to a poor TB control program performance at the community level, which leads to a poor case detection and treatment outcome, and may ultimately contribute to an increased number of treatment failure cases and the emergence and transmission of drug-resistant TB. A previous study showed that a high proportion of defaulters was reported in the Tigray Region compared to other regions of Ethiopia [13]. In addition, another study in Tigray reported a low level of knowledge about the cause of TB and the consequences of a poor treatment adherence to its treatment [69]. Besides, a recent assessment report in Tigray Region indicates that only 21.7% of household contacts were screened for TB by health extension workers [70], which are by far lower than the target stated by the national TB program to screen all household contacts [71] and WHO target of greater than 90% household contacts to be screened for TB [3].

Several factors have contributed to the development and transmission of drug-resistant TB. Genetic diversities of drug-resistant isolates might be attributable to some host and environmental factors besides strain evolution in different geographic regions [72–75]. Drug resistance in MTB arises at a low frequency of spontaneous chromosomal mutations and inconsistent drug supply, suboptimal prescription and poor patient adherence [73, 74]. In particular, patients who have a previous TB treatment history such as treatment failures, defaulters or relapse cases are at a greater risk of developing MDR-TB [5, 75, 76]. The WHO underscores the importance of the proper management of MDR-TB to help address its global threat to human beings. To help alleviate this threat, the WHO has recommended five priority actions, including the prevention of the development of MDR-TB through a high-quality treatment of drug-susceptible TB, expanding the rapid testing and detection of drug-resistant TB, thereby providing immediate access to effective treatment and proper care, the prevention of transmission through infection control and an increase in political commitment and financing. High TB-burden countries like Ethiopia should be committed to optimally implementing these actions to reduce the emergence and transmission of drug-resistant TB in Tigray and all other regions of the country.

### Limitations of the study

One of the limitations of this study was that non-MDR-TB isolates were not tested for second-line anti-TB drugs, as other studies found that such types of isolates were highly resistant to second-line anti-TB drugs [44]. The other limitation was that extra-pulmonary patients were not included in the study. Hence, the authors recommend further study on the overall study of the drug susceptibility pattern of first- and second line anti-TB drugs for non-MDR and MDR-TB from pulmonary and extra-pulmonary patients, in order to obtain full information on the magnitude of DR-TB in the study area.

## Conclusion

The magnitude of MDR-TB observed among new and previously treated patients is very alarming, which calls for an urgent need for intervention. The high proportion of MDR-TB among newly diagnosed cases indicates an ongoing transmission, which suggests the need for an enhanced TB control program performance to interrupt transmission. The increased proportion of MDR-TB among previously treated cases indicates a need for better patient management to help prevent the evolution of drug resistance. The associated factors of this study indicate the need for consideration of these predisposing factors in the prevention and intervention program of the Region. Overall, the findings highlight the importance of strengthening the Regional TB Control Program to detect and provide early appropriate treatment and follow-up for TB cases. Assessing the TB control program performance gaps in the region, and an optimal implementation of the five WHO-recommended priority actions for the management of drug-resistant TB, is imperative to reduce the current high MDR-TB burden in the study region.

## Supporting information

S1 Data (XLSX)

S1 File Questionnaire. (PDF)

## Acronym

DR-TB: drug resistant tuberculosis
FMOH: Federal Ministry of Health of Ethiopia
INH: Isoniazid
LPA: Line probe assay
MDR-TB: Multidrug-resistant tuberculosis
MTB: *Mycobacterium tuberculosis*
Pre-XDR-TB: Pre-extensively drug-resistant tuberculosis
PTB: Pulmonary tuberculosis
RIF: Rifampicin
WHO: World Health Organization

## References

[1] World Health Organisation. Global Tuberculosis Report 2018 [Internet]. Geneva; 2018. Available from: https://reliefweb.int/sites/reliefweb.int/files/resources/9789241565646-eng.pdf

[2] Global Tuberculosis Report [Internet]. Vol. 312, World Health Organization. Geneva; 2017. Available from: https://reliefweb.int/sites/reliefweb.int/files/resources/9789241565516-eng.pdf

[3] World Health Organization. Global Tuberculosis report 2018 [Internet]. Geneva; 2019. Available from: https://apps.who.int/iris/bitstream/handle/10665/329368/9789241565714-eng.pdf?ua=1

[4] Esmael A, Ali I, Agonafir M, Endris M, Getahun M et al Drug Resistance Pattern of Mycobacterium tuberculosis in Eastern Amhara Regional State, Ethiopia. J Microb Biochem Technol [Internet]. 2014;6:075–9. Available from: 10.4172/1948-5948.1000125

[5] Biadglegne F, Sack U, Rodloff AC. Multidrug-resistant tuberculosis in Ethiopia: Efforts to expand diagnostic services, treatment and care. Antimicrob Resist Infect Control. 2014;3(31):1–10. 10.1186/2047-2994-3-31 PMC4328048 25685333

[6] Eshetie S, Gizachew M, Dagnew M, Kumera G, Woldie H, Ambaw F, et al Multidrug resistant tuberculosis in Ethiopian settings and its association with previous history of anti-tuberculosis treatment: A systematic review and meta-analysis. BMC Infect Dis. 2017;17(219):1–12. 28320336 10.1186/s12879-017-2323-y PMC5360058

[7] Asgedom SW, Teweldemedhin M, Gebreyesus H. Prevalence of Multidrug-Resistant Tuberculosis and Associated Factors in Ethiopia: A Systematic Review. J Pathog [Internet]. 2018;2018:1–8. Available from: https://www.hindawi.com/journals/jpath/2018/7104921/10.1155/2018/7104921 PMC5903304 29850257

[8] Mulisa G, Workneh T, Hordofa N, Suaudi M, Abebe G, Jarso G. Multidrug-resistant Mycobacterium tuberculosis and associated risk factors in Oromia Region of Ethiopia. Int J Infect Dis [Internet]. 2015;39:57–61. Available from: 10.1016/j.ijid.2015.08.013 26327121

[9] Brhane M, Kebede A, Petros Y. Molecular detection of multidrug-resistant tuberculosis among smear-positive pulmonary tuberculosis patients in Jigjiga town, Ethiopia. Infect Drug Resist. 2017;10:75–83. 10.2147/IDR.S127903 28331348 PMC5352243

[10] Tadesse M, Aragaw D, Dimah B, Efa F, Abdella K, Kebede W, et al Drug resistance-conferring mutations in Mycobacterium tuberculosis from pulmonary tuberculosis patients in Southwest Ethiopia. Int J Mycobacteriology. 2016;5(2):185–91. 10.1016/j.ijmyco.2016.02.009 27242230

[11] Mekonnen D, Admassu A, Mulu W, Amor A, Benito A, Gelaye W, et al Multidrug-resistant and heteroresistant Mycobacterium tuberculosis and associated gene mutations in Ethiopia. Int J Infect Dis [Internet]. 2015 10 1 [cited 2018 Dec 13];39:34–8. Available from: https://www.sciencedirect.com/science/article/pii/S1201971215001472 10.1016/j.ijid.2015.06.013 26119857

[12] Mesfin EA, Beyene D, Tesfaye A, Admasu A, Addise D, Amare M, et al Drug-resistance patterns of mycobacterium tuberculosis strains and associated risk factors among multi drug-resistant tuberculosis suspected patients from Ethiopia. PLoS One. 2018;13(6):1–16. 10.1371/journal.pone.0197737 PMC5986145 29864118

[13] Eshetie S, Gizachew M, Alebel A, Van Soolingen D. Tuberculosis treatment outcomes in Ethiopia from 2003 to 2016, and impact of HIV co-infection and prior drug exposure: A systematic review and meta-analysis. PLoS One [Internet]. 2018;13(3):1–18. Available from: 10.1371/journal.pone.0194675 PMC5858841 29554144

[14] Pradipta IS, Van’T Boveneind-Vrubleuskaya N, Akkerman OW, Alffenaar JWC, Hak E. Treatment outcomes of drug-resistant tuberculosis in the Netherlands, 2005–2015. Antimicrob Resist Infect Control. 2019;8(1):1–12. 31338162 10.1186/s13756-019-0561-z PMC6626402

[15] Federal Democratic Republic of Ethiopia Central Statistical Agency Population Projection of Ethiopia for All Regions At Wereda Level from 2014–2017. Addis Ababa; 2017.

[16] Ministry of Health of Ethiopia. National Guidelines for HIV & AIDS Care and Treatment. 2017.

[17] Ministry of Health of Ethiopia. National comprehensive tuberculosis, leprosy and TB / HIV training manual for health care workers. Participants’ manual. [Internet]. ADDIS ABABA 1; 2016. Available from: https://www.slideshare.net/suleymanfantahun/new-ethiopian-tb-guildline-november-2016

[18] Cepheid. GeneXpert® operator’s manual and the Xpert® MTB/RIF protocols (CD-ROM) for comprehensive operating instructions, including important warnings relating to operator safety. Vol. 39, Animal Genetics. 2013.

[19] Edition F. Mycobacteriology Laboratory Manual Editor: In: first. 2014.

[20] GenoType MTBDRplus VER 2.0. Molecular genetic assay for identification of the M. tuberculosis complex and its resistance to Rifampicin and Isoniazid from clinical specimens and cultivated samples. Hain Lifescience, Nehren, Germany; 2015.

[21] Olson S, English R, Claiborne A. Institute of Medicine (US) Forum on Drug Discovery, Development, and Translation; Russian Academy of Medical Science. The New Profile of Drug-Resistant Tuberculosis in Russia: A Global and Local Perspective: Summary of a Joint Workshop. [Internet]. Transmission and Infection Control of Drug-Resistant TB. Washington (DC): National Academies Press (US); 2011. Available from: https://www.ncbi.nlm.nih.gov/books/NBK62463/22013610

[22] Sinshaw W, Kebede A, Bitew A, Tesfaye E, Tadesse M, Mehamed Z, et al Prevalence of tuberculosis, multidrug resistant tuberculosis and associated risk factors among smear negative presumptive pulmonary tuberculosis patients in Addis Ababa, Ethiopia. BMC Infect Dis. 2019;19(641):1–15. 31324227 10.1186/s12879-019-4241-7 PMC6642575

[23] Demile B., Zenebu A., Shewaye H et al Risk factors associated with multidrug-resistant tuberculosis (MDR-TB) in a tertiary armed force referral and teaching hospital, Ethiopia. BMC Infect Dis. 2018;18(249):1–10. 29855354 10.1186/s12879-018-3167-9 PMC5984406

[24] Mekonnen F, Tessema B, Moges F, Gelaw A, Eshetie S, Kumera G. Multidrug resistant tuberculosis: Prevalence and risk factors in districts of metema and west armachiho, Northwest Ethiopia. BMC Infect Dis [Internet]. 2015;15(461):2–7. Available from: 10.1186/s12879-015-1202-7 26503269 PMC4624367

[25] Alelign A, Zewude A, Mohammed T, Tolosa S, Ameni G, Petros B. Molecular detection of Mycobacterium tuberculosis sensitivity to rifampicin and isoniazid in South Gondar Zone, northwest Ethiopia. BMC Infect Dis. 2019;19(343):1–8. 31023267 10.1186/s12879-019-3978-3 PMC6485134

[26] Hamusse SD, Teshome D, Hussen MS, Demissie M, Lindtjørn B. Primary and secondary anti-tuberculosis drug resistance in Hitossa District of Arsi Zone, Oromia Regional State, Central Ethiopia. BMC Public Health [Internet]. 2016;16(593):1–10. Available from: 10.1186/s12889-016-3210-y 27430266 PMC4950499

[27] Hu Y, Hoffner S, Wu L, Zhao Q, Jiang W. Prevalence and genetic characterization of second-line drug- resistant and extensively drug-resistant Mycobacterium tuberculosis in rural China. Antimicrob Agents Chemother. 2013;57(8):3857–63. 10.1128/AAC.00102-13 23733477 PMC3719720

[28] Yonge SA, Otieno MF, Sharma RR, Nteka SS. Drug susceptibility patterns of Mycobacterium tuberculosis isolates from tuberculosis patients in Coastal Kenya. J Tuberc Res. 2017;5:201–19.

[29] Hoza AS, Abubakar S. Hoza Sayoki G.M. Mfinanga BK onig. Anti-TB drug resistance in Tanga, Tanzania: A cross sectional facility-base prevalence among pulmonary TB patients. Asian Pac J Trop Med. 2015;8(11):907–13. 10.1016/j.apjtm.2015.10.014 26614989

[30] Vidyaraj CK, Chitra A, Smita S, Muthuraj M, Govindarajan S, Usharani B, et al Prevalence of rifampicin-resistant Mycobacterium tuberculosis among human-immunodeficiency-virus-seropositive patients and their treatment outcomes. J Epidemiol Glob Health [Internet]. 2017;7(4):289–94. Available from: 10.1016/j.jegh.2017.09.002 29110871 PMC7384569

[31] Nguyen HB, Nguyen NV, Thi H, Tran G, Nguyen HV, Thi Q, et al Prevalence of resistance to second-line tuberculosis drug among multidrug-resistant tuberculosis patients in Viet Nam, 2011. JWPSAR. 2016;7(2):35–40. 10.5365/WPSAR.2016.7.2.002 PMC4957611 27508089

[32] Villegas L, Otero L, Sterling TR, Huaman MA. Prevalence, risk factors, and treatment outcomes of Isoniazid- and Rifampicin- mono-resistant pulmonary tuberculosis in Lima, Peru. PLoS One [Internet]. 2016;11(4):1–11. Available from: 10.1371/journal.pone.0152933 PMC4821555 27045684

[33] Lv X, Lu X, Shi X. Prevalence and risk factors of multi-drug resistant tuberculosis in Dalian, China. J Inte Medi Res. 2017;45(6):1779–1786. 10.1177/0300060516687429 PMC5805195 28345426

[34] Liu Q, Zhu L, Shao Y, Song H, Li G, Zhou Y, et al Rates and risk factors for drug resistance tuberculosis in Northeastern China. BioMed Cent Ltd. 2013;13(1171):1–7. 10.1186/1471-2458-13-1171 PMC3878749 24330553

[35] Kigozi E, Kasule GW, Musisi K, Lukoye D, Kyobe S, Katabazi FA, et al Prevalence and patterns of rifampicin and isoniazid resistance conferring mutations in Mycobacterium tuberculosis isolates from Uganda. PLoS One. 2018;13(5):1–17. 10.1371/journal.pone.0198091 PMC5976185 29847567

[36] Wei-Juin Su, Jia-Yih Feng, Chin-Chou Huang R-PP. Increasing drug resistance of Mycobacterium tuberculosis isolates in a medical center in Northern Taiwan. J Formos Med Assoc. 2008;107(3):259–64. 10.1016/S0929-6646(08)60145-X 18400612

[37] Seyoum B, Demissie M, Worku A, Bekele S, Aseffa A. Prevalence and drug resistance patterns of Mycobacterium tuberculosis among new smear positive pulmonary tuberculosis patients in Eastern Ethiopia. Tuberc Res Treat [Internet]. 2014;2014:1–7. Available from: http://www.hindawi.com/journals/trt/2014/753492/10.1155/2014/753492 PMC4009208 24834351

[38] Yimer SA, Agonafir M, Derese Y, Sani Y, Bjune GA, Holm-Hansen C. Primary drug resistance to anti-tuberculosis drugs in major towns of Amhara region, Ethiopia. Apmis. 2012;120(6):503–9. 10.1111/j.1600-0463.2011.02861.x 22583363

[39] Warren RM. The epidemiology, pathogenesis, transmission, diagnosis, and management of multidrug-resistant, extensively drug-resistant, and incurable tuberculosis. Lancet Respir Med Comm. 2017;5(4):291–360. 10.1016/S2213-2600(17)30079-6 28344011

[40] Abdella K, Abdissa K, Kebede W, Abebe G. Drug resistance patterns of Mycobacterium tuberculosis complex and associated factors among retreatment cases around Jimma, Southwest Ethiopia. BMC Public Health [Internet]. 2015;15(599):1–7. Available from: 10.1186/s12889-015-1955-3 26135909 PMC4489121

[41] Abate D, Tedla Y, Meressa D, Ameni G. Isoniazid and rifampicin resistance mutations and their effect on second-line anti-tuberculosis treatment, Addis Ababa, Ethiopia. INT J TUBERC LUNG DIS. 2014;18(8):946–51. 10.5588/ijtld.13.0926 25199009

[42] Id AS, Gelaw B, Gebreyes W, Robinson R, Wang S, Tessema B. The burden of pre-extensively and extensively drug-resistant tuberculosis among MDR-TB patients in the Amhara region, Ethiopia. PLoS One [Internet]. 2020;15(2):1–13. Available from: 10.1371/journal.pone.0229040 PMC7018133 32053661

[43] Oudghiri A, Karimi H, Chetioui F, Zakham F, Bourkadi JE, Elmessaoudi MD, et al Molecular characterization of mutations associated with resistance to second-line tuberculosis drug among multidrug-resistant tuberculosis patients from high prevalence tuberculosis city in Morocco. BMC Infect Dis. 2018;18(98):1–8. 29486710 10.1186/s12879-018-3009-9 PMC5830342

[44] Jain A, Prasad R. Pre-XDR & XDR in MDR and Ofloxacin and Kanamycin resistance in non-MDR Mycobacterium tuberculosis isolates. J Tuberc Elsevier. 2012;92(5):404–6. 10.1016/j.tube.2012.05.010 22789499

[45] Patel SM, Patel MH, Soni ST, Vegad MM. Second-line drug resistance patterns among patients with multidrug-resistant tuberculosis of Gujarat, western India. Int J Med Sci Public Heal. 2015;4(5):639–41.

[46] Atif M, Bashir A, Ahmad N, Fatima RK, Saba S, Scahill S. Predictors of unsuccessful interim treatment outcomes of multidrug resistant tuberculosis patients. BMC Infect Dis. 2017;17(655):1–12. 28962599 10.1186/s12879-017-2746-5 PMC5622487

[47] Baya B, Achenbach CJ, Kone B, Toloba Y, Dabitao DK, Diarra B, et al Clinical risk factors associated with multidrug-resistant tuberculosis (MDR-TB) in Mali. Int J Infect Dis [Internet]. 2019 4 1;81:149–55. Available from: 10.1016/j.ijid.2019.02.004 30772470 PMC6481646

[48] Zhao P, Li XJ, Zhang SF, Wang XS, Liu CY. Social behaviour risk factors for drug resistant tuberculosis in mainland china: A meta-analysis. J Int Med Res. 2012;40(2):436–45. 10.1177/147323001204000205 22613404

[49] Workicho A, Kassahun W, Alemseged F. Risk factors for multidrug resistant tuberculosis among tuberculosis patients: a case-control study at St. Peter’s TB Specialized Hospital, Addis Ababa, Ethiopia. Infect Drug Resist. 2017;10:91–6. 10.2147/IDR.S126274 28331350 PMC5357068

[50] Liang L, Wu Q, Gao L, Hao Y, Liu C, Xie Y, et al Factors contributing to the high prevalence of multidrug-resistant tuberculosis: a study from China. Thorax BMJ. 2012;67(7):632–8. 10.1136/thoraxjnl-2011-200018 22403070

[51] Zhang C, Wang Y, Shi G, Han W, Zhao H, Zhang H, et al Determinants of multidrug-resistant tuberculosis in Henan province in China: a case control study. BMC Public Health. 2016;16(1):1–8. 26775263 10.1186/s12889-016-2711-z PMC4715352

[52] Demelash Assefa, Berhanu Seyoum LO. Determinants of multidrug-resistant tuberculosis in Addis Ababa, Ethiopia. Infect Drug Resist. 2017;10:209–13. 10.2147/IDR.S134369 28744149 PMC5513846

[53] Rifat M, Hall J, Oldmeadow C, Husain A, Hinderaker SG, Milton AH. Factors related to previous tuberculosis treatment of patients with multidrugresistant tuberculosis in Bangladesh. BMJ Open. 2015;5(9):1–9. 10.1136/bmjopen-2015-008273 PMC4563275 26351185

[54] Merza MA, Farnia P, Tabarsi P, Khazampour M, Masjedi MR, Velayati AA. Anti-tuberculosis drug resistance and associated risk factors in a tertiary level TB centre in Iran: A retrospective analysis. J Infect Dev Ctries. 2011;5(7):511–9. 10.3855/jidc.1259 21795819

[55] Hirpa S, Medhin G, Girma B, Melese M, Mekonen A, Suarez P, et al Determinants of multidrug-resistant tuberculosis in patients who underwent first-line treatment in Addis Ababa: A case control study. J BMC Public Heal [Internet]. 2013;13(782):2–9. Available from: BMC Public Health 10.1186/1471-2458-13-782 PMC4015150 23981845

[56] Silva DR, Muñoz-Torrico M, Duarte R, Galvão T, Bonini EH, Arbex FF, et al Risk factors for tuberculosis: Diabetes, smoking, alcohol use, and the use of other drugs. J Bras Pneumol. 2018;44(2):145–52. 10.1590/s1806-37562017000000443 29791552 PMC6044656

[57] El Hamdouni M, Bourkadi JE, Benamor J, Hassar M, Cherrah Y, Ahid S. Treatment outcomes of drug resistant tuberculosis patients in Morocco: Multi-centric prospective study. BMC Infect Dis. 2019;19(316):1–7. 30975090 10.1186/s12879-019-3931-5 PMC6458640

[58] Chuchottaworn C, Thanachartwet V, Sangsayunh P, Zar T. Risk factors for multidrug resistant tuberculosis among patients with pulmonary tuberculosis at the Central chest institute of Thailand. PLoS One [Internet]. 2015;10(10):1–17. Available from: 10.1371/journal.pone.0139986 PMC4596622 26444421

[59] Colijn C, Cohen T, Ganesh A, Murray M. Spontaneous emergence of multiple drug resistance in tuberculosis before and during therapy. PLoS One. 2011;6(3). 10.1371/journal.pone.0018327 PMC3068161 21479171

[60] Singla R, Bharty SK, Gupta UA, Khayyam KU, Vohra V, Singla N, et al Sputum smear positivity at two months in previously untreated pulmonary tuberculosis patients. Int J Mycobacteriology. 2013;2(4):199–205. 10.1016/j.ijmyco.2013.08.002 26786122

[61] Gunda DW, Nkandala I, Kavishe GA, Kilonzo SB, Kabangila R, Mpondo BC. Prevalence and Risk Factors of Delayed Sputum Conversion among Patients Treated for Smear Positive PTB in Northwestern Rural Tanzania: A Retrospective Cohort Study. J Trop Med. 2017;2017:1–5. 10.1155/2017/5352906 PMC5485336 28694828

[62] Wells CD, Cegielski JP, Nelson LJ, Laserson KF, Holtz TH, Finlay A, et al HIV Infection and Multidrug‐Resistant Tuberculosis—The Perfect Storm. J Infect Dis. 2007;196:S86–107. 10.1086/518665 17624830

[63] Esmael A, Ibrahim Ali, Mulualem Agonafir, Mengistu Endris, Muluworke Getahun ZY and KD. Drug resistance pattern of Mycobacterium tuberculosis in Eastern Amhara Regional State, Ethiopia. J Microb Biochem Technol [Internet]. 2014;06:75–9. Available from: https://www.omicsonline.org/open-access/drug-resistance-pattern-of-mycobacterium-tuberculosis-in-eastern-amhara-regional-state-ethiopia-1948-5948.1000125.php?aid=22389

[64] Mehari K, Asmelash T, Hailekiros H, Wubayehu T, Godefay H, Araya T, et al Prevalence and Factors Associated with Multidrug-Resistant Tuberculosis (MDR-TB) among Presumptive MDR-TB Patients in Tigray Region, Northern Ethiopia. Can J Infect Dis Med Microbiol. 2019;2019:1–8. 10.1155/2019/2923549 PMC6754863 31583034

[65] Fikre A, Tewelde T, Shaweno T. Determinants of multi drug resistant tuberculosis among tuberculosis patients in Southern Ethiopia: A Case Control Study. J Med Bacteriol [Internet]. 2019 2 19;8(1–2):1–12. Available from: http://jmb.tums.ac.ir/index.php/jmb/article/view/390

[66] Sisay S, Mengistu B, Erku W, Woldeyohannes D. Directly Observed Treatment Short-course (DOTS) for tuberculosis control program in Gambella Regional State, Ethiopia: ten years experience. 2014; 10.1186/1756-0500-7-44 PMC3905666 24444379

[67] Datiko DG, Lindtjørn B. Health extension workers improve tuberculosis case detection and treatment success in southern Ethiopia: A community randomized trial. PLoS One. 2009;4(5):e5443 10.1371/journal.pone.0005443 19424460 PMC2678194

[68] Sebastian MS, Lemma H. Efficiency of the health extension programme in Tigray, Ethiopia: A data envelopment analysis. BMC Int Health Hum Rights. 2010;10(16):1–8. 20546597 10.1186/1472-698X-10-16 PMC2893104

[69] Adane K, Spigt M, Johanna L, Noortje D, Abera SF, Dinant GJ. Tuberculosis knowledge, attitudes, and practices among northern Ethiopian prisoners: Implications for TB control efforts. PLoS One. 2017;12(3):e0174692 10.1371/journal.pone.0174692 28358877 PMC5373603

[70] Gebretnsae H, Ayele BG, Hadgu T, Haregot E, Gebremedhin A, Michael E, et al Implementation status of household contact tuberculosis screening by health extension workers: Assessment findings from programme implementation in Tigray region, northern Ethiopia. BMC Health Serv Res. 2020;20(1):1–7. 10.1186/s12913-020-4928-x PMC6995142 32005226

[71] Federal Ministry of Health Ethiopia, TB. B/HIV Prevention and Control Tuberculosis, Leprosy and Programme, manual Fourth Edition. Addis Ababa; 2008.

[72] Maharjan B, Nakajima C, Isoda N, Thapa J, Poudel A, Shah Y, et al Genetic diversity and distribution dynamics of multidrug-resistant Mycobacterium tuberculosis isolates in Nepal. Sci Rep. 2018;8(1):1–10. 10.1038/s41598-017-17765-5 30413747 PMC6226479

[73] Jou R, Chen HY, Chiang CY, Yu MC, Su IJ. Genetic diversity of multidrug-resistant Mycobacterium tuberculosis isolates and identification of 11 novel rpoB alleles in Taiwan. J Clin Microbiol. 2005;43(3):1390–4. 10.1128/JCM.43.3.1390-1394.2005 15750115 PMC1081303

[74] Mbugi E V., Katale BZ, Streicher EM, Keyyu JD, Kendall SL, Dockrell HM, et al Mapping of Mycobacterium tuberculosis Complex Genetic Diversity Profiles in Tanzania and Other African Countries. PLoS One. 2016;11(5):: e0154571 10.1371/journal.pone.0154571 27149626 PMC4858144

[75] Gagneux S, Burgos M V., DeRiemer K, Enciso A, Muñoz S, Hopewell PC, et al Impact of bacterial genetics on the transmission of isoniazid-resistant Mycobacterium tuberculosis. PLoS Pathog. 2006;2(6):0603–10. 10.1371/journal.ppat.0020061 PMC1479046 16789833

[76] Zhang Y. WWY. Mechanisms of drug resistance in Mycobacterium tuberculosis. INT J TUBERC LUNG DIS. 2009;13(11):1320–30. 19861002

