# Correction: Characteristics of pulmonary multidrug-resistant tuberculosis patients in Tigray Region, Ethiopia: A cross-sectional study

**DOI:** 10.1371/journal.pone.0258457

**Published:** 2021-10-06

**Authors:** Letemichael Negash Welekidan, Eystein Skjerve, Tsehaye Asmelash Dejene, Mengistu Welday Gebremichael, Ola Brynildsrud, Angelika Agdestein, Girum Tadesse Tessema, Tone Tønjum, Solomon Abebe Yimer

The affiliation for the ninth author is incorrect. Solomon Abebe Yimer is not affiliated with #5. An additional affiliation is missing for this author. Solomon Abebe Yimer is also affiliated with Coalition for Epidemic Preparedness Innovations, Oslo, Norway.
