# Supplementary material for: Characteristics of pulmonary multidrug-resistant tuberculosis patients in Tigray Region, Ethiopia: A cross-sectional study
Source: PLoS One. 2020 Aug 14;15(8):e0236362. doi: 10.1371/journal.pone.0236362 (PMC7428183; doi:10.1371/journal.pone.0236362)
Supplement: S1 File — (PDF) [file pone.0236362.s002.pdf]

INFORMED CONSENT FORM (≥18 years old)

By signing below, I confirm the following:

- I have been given oral and written information about the proposed study; have read/been read and understood the information given
- I have had sufficient time to consider participation in the study and have had the opportunity to ask questions and all my questions have been answered satisfactorily
- I know that participation in this project is voluntary, and I have understood the content and the objective of the study
- I am further aware that the information and specimen I will provide will only be utilized in the proposed study
- I recognize that all the information regarding myself will be kept completely confidential, and I know that I can quit any time from this study without providing any reason for doing so
- I know that sputum or culture (*M. tuberculosis*) will be transferred to Norway, veterinary institute for advanced laboratory analyses.

It is therefore with full understanding of the situation that I am giving my voluntary informed consent

Name_________________________Signature_________Place______Date_______________

Witnesses name (for those who do not read and write)

1. Name _____________________________________Signature _______________________

Name of the person who take consent ____

signature________________Place___________________________Date_______________

INFORMED ASSENT FORM (15-17 Years of Age)

By signing below, I confirm the following:

- I have been given oral and written information about the proposed study; have read/been read and understood the information given.
- I have had sufficient time to consider participation of my child/relative in the study and have had the opportunity to ask questions and all my questions have been answered satisfactorily.
- I know that participation in this project is voluntary, and I have understood the content and the objective of the study.
- I am further aware that the information and specimen my child /relative will provide will only be utilized in the proposed study
- I recognize that all the information regarding my child/relative will be kept completely confidential, and I know that I can quit any time from this study without providing any reason for doing so.
- I know that sputum or culture (M. *tuberculosis*) will be transferred to Norway, Veterinary institute for advanced laboratory analyses.

It is therefore with full understanding of the situation that I am giving my voluntary informed consent

Name_________________________Signature____________Place___________Date____

Name of family/next of kin____________________ Signature_________ Place_____ Date____

Witnesses name (for those who do not read and write)

1. Name ______________________________________Signature _____________

Name of the person who take consent ____________________________________

signature______________Place_________ _____________Date_________________

Questionnaire for patients

Date of data collection:_____E.C

Name of data collector and signature:_______________________Mobile:_________________

Name of Health institution (Health center /Hospital): ___________________Zone:__________

Patient Card number:_______sputum Code (assigned by data collector):________

Gen X-pert Results

A. **MTB Detected, Rif not detected or R susceptible____________**
B. **MTB Detected, RR_____________**

TB enrolment

TB register number: ___________MDR register number: ____________

Demographic and Socio-economic data

1. Patient's Full Name____
2. Where do you live (**Residence**)?
   A. Urban
   B. Rural

   2.1. Location: Block number :__________________________________
   Kushet/ketena_______________________Tabia/Kebelle:_____
   Wereda/district:__________________________Zone:_____________
   mobile Number__________

3. Sex
   A. Male
   B. Female
4. **Age**:________
5. Marital status?**(for patients≥18)**
   A. Single
   B. Married
   C. Widowed
   D. Divorced
   E. Not applicable

   5.1.If married women ≥**18**, number of births/child:________
   5.2.If married women ≥**18**, current condition
   A. Pregnant
   B. Lactating

   C. None pregnant and none lactating

6. What is your family size? __________________
7. What is your monthly family income (in birr)?
   A. <500
   B. 500-2000
   C. >2000
   D. No means of income
8. What is the highest level of education you have attained at present?
   A. Illiterate
   B. 1-8 grades
   C. 9-12 grades
   D. Diploma and above
9. What is your current occupation?
   A. House wife
   B. Farmer
   C. Self employed
   D. Government employee
   E. Student
   F. No work

Clinical Presentation

10. Would you please tell me if you have one of the following symptoms currently?
    A. Weight loss
    B. Chest pain when breathing or coughing
    C. Coughing up sputum > 2 weeks
    D. Hemoptysis
    E. Shortness of breath
    F. Intermittent fever
    G. Night sweats
    H. Loss of appetite
    I. Fatigue and malaise
    J. Other___________

11. When do you start coughing?_______

TB history and related disease

12. Type of TB case
    A. New (never treated before for ≥1 month)
    B. Relapse
    C. Return after default

D. Failure after treatment

13. Do you have any history of TB patient in your family?
    A. Yes
    B. No
14. Do you have any close contact with TB patient or chronically coughing patient?
    A. Yes
    B. No
15. If yes, for how long do you had a contact?_________
16. Have you any close contact with MDR patient?
    A. Yes
    B. No
17. If yes, for how long do you had a contact?_________
18. History of diabetes mellitus?
    A. Yes
    B. No
19. HIV status
    A. Positive
    B. Negative
20. Nutritional status/Anthropometric measurement
    A. Weight in Kg________
    B. Height in cm________
21. Are you currently taking treatment for chronic disease?
    A. Yes
    B. No
22. If yes, type of treatment/type of chronic disease:________________

Behavior of the patient (≥18 years)

23. Do you have the habit of **alcohol taking**?
    A. Yes
    B. No
24. If yes, how many time per week?__________
    A. 1-2/week
    B. 3-5/ week
    C. 6-7 /week
    D. ≥2 /day
25. What habit do you have?
    A. Khat chewing

B. Cigarette smoking

C. Others __________

D. No habit

26. History of prison?

A. Yes

B. No

27. If yes, for how long you have been in prison?___________________

- **Date of release to regional laboratory________________________E.C**

Laboratory data recording format for patients

Laboratory results:

- ✓ Date of specimen received by THRL: ____/___/___ E.C

Name of investigator: _____________Mobile:______

Laboratory sputum ID: ___________, Culture Number: _____________

1^0 smear	Media Smear	Blood agar	Capila

TB Culture results

Neg	Pos	conta	NTM	Method

Date reported: ____/____/____Name and Signature of expert _______________________

Results of *M. tuberculosis* drug susceptibility test

✓**Molecular method used (LPA)**

Result	1st line drugs					2nd line drugs							Other	
	H	R	S	E	PZA	OFX	LFT	MFX	AMK	CAP	KAN	VIO		

Legend: H= Isoniazid, R= Rifampicin, PZA= Pyrazinamide, E=Ethambutol, S= Streptomycin, Ofx= Ofloxacin, Lft=levofloxacin, Mfx= Moxifloxacin, AMK= Amikacin, CAP= Capromycin, KAN=kanamycin, Vio=viomycin, S = Sensitive, R = Resistant, C = Contaminated, ND = Not done

Comment: ____

Date reported: ____/____/____Name and Signature of expert _______________________
