# Supplementary material for: Characteristics of pulmonary multidrug-resistant tuberculosis patients in Tigray Region, Ethiopia: A cross-sectional study
Source: PLoS One. 2020 Aug 14;15(8):e0236362. doi: 10.1371/journal.pone.0236362 (PMC7428183; doi:10.1371/journal.pone.0236362)
Supplement: Attachment — Submitted filename: Response to Reviewers.docx [file pone.0236362.s003.docx]

# Authors’ reply Academic Editor: Frederick Quinn PLOS ONE

# Dear Editor

We would like to thank the reviewers for their in-depth comments and suggestions. The comments have enabled us to improve the quality of the manuscript (PONE-D-20-06975). In this authors’ reply, we have tried as much as possible to give a point- by- point reply for each reviewer’s comments. Reviewer’s comments are written in black text and authors reply is written in red text. The uploaded files include “revised manuscript with tack changes” using red colored text showing all changes made (deleted paragraphs, sentences, phrases and tables) and a clean version with file name “Manuscript” with no colored text have been uploaded.

If you would like to make changes to your financial disclosure, please include your updated statement in your cover letter.

Thank you very much for your suggestion

We have modified the name of the funder and updated the statement in the cover letter.

To enhance the reproducibility of your results, we recommend that if applicable you deposit your laboratory protocols in [protocols.io](http://protocols.io), where a protocol can be assigned its own identifier (DOI) such that it can be cited independently in the future. For instructions see: <http://journals.plos.org/plosone/s/submission-guidelines#loc-laboratory-protocols>
Thank you for your suggestion

The suggestion is very nice but we have used the standard operating procedures (SOP) of the laboratory where we did the laboratory procedures. We do not have my own SOP. If it is necessary to provide the SOPs of the laboratory, we can do it.

1. Please ensure that your manuscript meets PLOS ONE's style requirements, including those for file naming. The PLOS ONE style templates can be found at
<https://journals.plos.org/plosone/s/file?id=wjVg/PLOSOne_formatting_sample_main_body.pdf> and
<https://journals.plos.org/plosone/s/file?id=ba62/PLOSOne_formatting_sample_title_authors_affiliations.pdf>

Thank you for your suggestion

According to your suggestion, the manuscript is checked for the PLOS ONE's style requirements.

2. We noticed you have some minor occurrence(s) of overlapping text with the following previous publication(s), which needs to be addressed:
<https://doi.org/10.1186/s12879-017-2389-6>
<https://doi.org/10.1155/2019/2923549>
<http://www.stoptb.org/wg/gli/assets/documents/gli_mycobacteriology_lab_manual_web.pdf>

Thank you for your suggestion. We have addressed the overlapping texts in the methods section.

3. We note that you have indicated that data from this study are available upon request. PLOS only allows data to be available upon request if there are legal or ethical restrictions on sharing data publicly. For information on unacceptable data access restrictions, please see <http://journals.plos.org/plosone/s/data-availability#loc-unacceptable-data-access-restrictions>.

Thank you for your suggestion. We have changed the availability of data statement from “data are available upon request” to “data are included within the manuscript”. We mentioned it in the cover letter.

4. We note that Figures 1 and 3 in your submission contain [map/satellite] images which may be copyrighted. All PLOS content is published under the Creative Commons Attribution License (CC BY 4.0), which means that the manuscript, images, and Supporting Information files will be freely available online, and any third party is permitted to access, download, copy, distribute, and use these materials in any way, even commercially, with proper attribution. For these reasons, we cannot publish previously copyrighted maps or satellite images created using proprietary data, such as Google software (Google Maps, Street View, and Earth). For more information, see our copyright guidelines: <http://journals.plos.org/plosone/s/licenses-and-copyright>.

Thank you for your suggestion. We appreciate your concern, but this map was developed by the authors by taking information from a standard map of the study region and developed the study area map. Geographical position system (GPS) readings were taken for the hospitals to indicate where each hospital is located in the region. If you believe this map has a limited contribution to the paper we can agree to exclude it.

Response to comments from Reviewer 1
This study attempted to describe the burden and factors associated with MDR-TB in Tigray, Ethiopia. I notified several things that should be clarified by the authors:

1. The study is a cross-sectional study, which is not adequate to analyse factor associated with MDR-TB. Temporality degree should be clearly defined between the factors and outcomes. Since the factors and outcome were identified at the one point in the cross-sectional study, temporality degree is not precise. I suggest changing the term “factors” to “characteristics” of MDR-TB patients.

Thank you for your suggestion

As you have mentioned it very well, cross-sectional study cannot address real risk factors. Hence, we have assessed associated factors with the development of MDR-TB by statistically comparing the participants that have specific factors and with those participants who do not have these factors in the development of MDR-TB. That is why we have used associated factors instead of risk factors. If you still think this must be changed, we will consider it to change from “factors” to “characteristics”.
2. I read that the study defined Tigray, Ethiopia, as the target population and sixth public hospitals were included as the source population in this study. The question is about the adequateness of the sample size. I read that there are many hospitals (private and public) in Tigray and around thousands of people were identified as suspected TB patients. How can you ensure 300 participants and the only public hospital included will represent the TB population in Tigray?

Comment accepted. During data collection, these hospitals were the only hospitals that started GeneXpert as the primary diagnostic technique to all TB suspected patients from the six main zones of the Region. These hospitals had catchment health institutions around them to send sputum samples to these hospitals for diagnosis of TB by the GeneXpert. Out of the 6922 TB suspected patients, only 300 patients were GeneXpert positive. Hence, we have a strong belief that these may represent the Region, even though it is very difficult to be 100% sure. In Tigray Region, only the public hospitals are serving as TB/MDR-TB diagnostic and treatment centers. We have also discussed the representatively of samples in the manuscript.

3. Although you explained the sample size calculation, it is still not clear for me. How you defined the sample size?. There is no sampling frame described and if we look at the figure 2, it only 4.2% suspected TB patient who had positive Gen expert. It is a very small proportion. In another side, your source population is the presumptive TB patients. Can you explain that?

Comment accepted. The rationale behind the size of the study was as follows: The source population consisted of all presumptive pulmonary PTB cases in the Tigray Region during the study period. The study population were all presumptive PTB cases who visited the selected health facilities in the region during the study period. As stated, a total of 6922 patients were screened. But the study participants were based on the inclusion criteria included PTB patients’ who were not under treatment and ≥15 years of age, who were GeneXpert-positive and could provide written informed consent, were included in the study. Overall, there are source population → study population → study participants (our sample) which were included in to the study based on the stated inclusion criteria. As described in the manuscript, the study depended more on the number of GeneXpert positives that the population as such. In the manuscript we state the following, hopefully in a clear way, where we also state the precision calculations given our assumptions could be met.

“The sample size was determined by taking the required minimum number of MDR-TB patients to be enrolled in the study, which was estimated to be between 30-40 cases. This was to establish a sample with sufficient number of MDR-TB isolates to allow a sufficient power to find different varieties of MDR-TB isolates. Based upon an expected level of 10% MDR-TB among TB patients, we aimed at recruiting at least 300 TB patients for the study. This would allow us to sample enough patients for the molecular studies, and also maintain a reasonable level of precision for the prevalence estimates. Without adjusting for clustering, the precision of the estimate would be at 6.7%-14.2% (95% CI, a relative precision of ± 3.3%).”

4. Can you explain more detail why your data have a high proportion of negative gen expert test? Can you provide detail information about the excluded participants? And how you deal with the representativeness of your data?

Comment accepted. The reason for the high proportion of GeneXpert negative from presumptive PTB patients is because of all presumptive PTB patients or patients with the signs and symptoms of TB were diagnosed by GeneXpert as the primary diagnostic technique. It is not expected to get high proportion of GeneXpert positive PTB patients from presumptive TB patients, as you know, there are other infections with similar sign and symptoms with TB, and some of the GeneXpert positive patients were excluded based on the exclusion criteria (patients <15 years of age, extra pulmonary TB and critically ill patients).

The six hospitals represent the six main zones of the Region. These hospitals were the only hospitals equipped with GeneXpert test as the primary diagnostic technique. That is why these hospitals were selected to represent the Region.
5. Please follow standard reporting for observational study (e.g. STROBE)

Thank you for your suggestion. The manuscript is organized based on the standard reporting for observational study following the STROBE checklist.
6. One of your inclusion criteria is “new PTB patient” (line 120), but in the result, you provide data about previously treated TB patients (table 2)?

Comment accepted. We have changed “new PTB patient” to “PTB patients who were not under treatment for TB during sputum collection” (line 142).
7. No clear information on how missing data was managed. Please describe it

A data missed by some respondents were traced back to the participants and completed the missed data. A few data which were not very important and missed by most respondents were omitted from the analysis as a whole. If important data were missed by some participants and we could not complete it by tracing back, we included only the respondents in the analysis as you can see from the tables 3 and 5. We have mentioned this information in the data entry and analysis section lines 267-271.
Abstracts
8. The significant factors should be described in the result of abstract

Comment accepted. We have added the significant factors like intermittent fever (AOR= 2.54, 95% CI= 1.21-5.4) and alcohol consumption (AOR= 0.28, 95% CI= 0.10-0.77) in the abstract result section (lines 48-51).

9. Subject criteria should be described

## Comment accepted. We have included the inclusion criteria for the study participants in the abstract method section (lines 37-39). Introduction 10. Line 92, reference? Adding evidence from another country is suggested. See PMID: 30394362, PMID:31338162.

Comment accepted. Reference is added to for the previous one and the suggested evidence from other country (PMID: 31338162) (line 113).
Method
11. Line 119, please explain the reason to choose the selected hospital.

Comment accepted. As one of the inclusion criteria was GeneXpert-positive PTB patients, these hospitals were selected based on the availability of GeneXpert diagnostic technique as the primary test for presumptive PTB patients from the main zones of the Region. This explanation is added in the revised manuscript line 156.
12. Line 122, the excluded participants should be reported in the result section? How many? and what is the reason?

Comment accepted. A total of 6322 presumptive PTB patients were excluded from the study because of the exclusion criteria stated. Primarily, they were GeneXpert negative. It is explained in the result section line 292-293.
13. Line 166, how many TB-HIV patient?

Comment accepted.

We have already mentioned the proportion of non-MDR-TB/HIV and MDR-TB/HIV co-infection in the result section. Now we have added the overall proportion of TB/HIV co-infection (33%) in the result section lines 320-321.
14. Line 236, in which category patient with drug-resistant but not MDR-TB (e.g. mono resistance, IH-R, S-R, any R, etc.)?

Comment accepted. In line 275, we have changed the phrase “drug susceptible” to “non-MDR-TB”. We have categorized the patients in to non-MDR-TB and MDR-TB patients to see the statistical difference among them in the factors associated with MDR-TB. More about the other categories e.g. mono resistance to INH, RIF and any resistance are already mentioned in the result section and in table-2.
Result
15. Line 264, it’s should be tested statistically, and the next is focus on the description only for the included participant

Comment accepted. We have deleted the sentence “No major difference in demographic factors was found between the initial 300 and the final 227 participants” from the result section. And for further analysis, only the 227 study participants are described.

16. Table 1 it’s difficult to understand. Please revise

Comment accepted. We have revised table-1.
17. Table 3-5 can be combined. Did you analyse it separately? It should be interpreted in combination.

Thank you for your suggestion. However, if the three tables were l be merged into one table, it would be more congested and might be difficult to understand clearly by the readers. That is why we put the information in three tables based on the characteristic of the associated factors. It is analyzed separately but interpreted combinedly under the associated factors section.
18. Line 331-343. Please ensure that you compare with the same duration and unit of measurement

Comment accepted. We have ensured that we have compared with the same duration and unit of measurement.
Discussion
19. Some findings are different from the global risk factors of MDR-TB (PMID: 30339803), can you explain? It will guide for the specific problem in Tigray as compare with others and give insight for developing specific intervention in Tigray.

Comment accepted. This reference (PMID: 30339803) is very important to explain our finding why it is different with other regions of Ethiopia and the global risk factors. We have explained in the discussion section (lines 461-463) and added a recommendation in the conclusions part accordingly (lines 509-510).

Response to comments from Reviewer 2:

The prevalence of MDR TB is found to be very high in new and retreatment cases in the Tigray region in Ethiopia. This is an important result from public health standpoint in terms of allocation of resources and need to intervene to limit spread. It would be relevant for authors to discuss in methods section why the six hospitals were selected from the region and if there is a possibility that the population served by the hospitals may have a higher prevalence or risk of MDR TB, such has presence of hotspots, high HIV prevalence, historically poor TB control, etc. It should be discussed in the discussion why the prevalence of MDR TB is higher in the region than in most of the other parts of the countries.

In the methods, authors should describe the standard of practice in these hospitals and the region before this survey. Do all patients get Xpert or culture? Or with this study, are the patients getting systematic DST? If so, is there posssiblity this could influence the prevalence that was found.

Comment accepted.

Method section

The hospitals were selected based on the availability of GeneXpert test only from the main zones of the Region without considering other factors (risk of high MDR-TB, high HIV, historically poor TB control, etc). The selected hospitals were the only hospitals with the GeneXpert test for diagnosis of all presumptive PTB patients during the study. These hospitals were giving diagnosis services for other neighboring health institutes that do not have GeneXpert test.

The standard practice of the hospitals was, 1^st^ testing of all presumptive PTB patients by a GeneXpert test, if the GeneXpert test result is susceptible TB, the patient is immediately started on 1^st^ line-TB treatment and if the result is RIF-R TB, the patient will be linked to MDR-TB clinic and immediately sputum sample used to be collected for culture before starting MDR-TB treatment.

For the purpose of this study, sputum samples were collected for culture and drug susceptibility testing from all consecutive GeneXpert positive PTB patients who fulfilled the inclusion criteria. This could not affect for the proportion of MDR-TB.

This information is described in the method section (lines 156-169).

Discussion Section

We have already tried to mention the possible reasons for the high proportion of MDR-TB in the study area compared with the other Regions of Ethiopia in the discussion section from lines 464-476 of the original manuscript. Besides, we have added now additional possible reason (lines 476-479) why it is higher than other studies in the country. Household contact tracing is the key strategy for the TB prevention program. However, “There is a recent assessment report that indicates in Tigray Region only 21.7% of household contacts of index TB case were screened for TB by health extension workers which are by far lower than the target stated by national TB screening program implementation to screen all household contacts by health extension workers and WHO target of greater than 90% household contacts of index TB case to be screened for TB”.
