# Supplementary material for: Characteristics of pulmonary multidrug-resistant tuberculosis patients in Tigray Region, Ethiopia: A cross-sectional study
Source: PLoS One. 2020 Aug 14;15(8):e0236362. doi: 10.1371/journal.pone.0236362 (PMC7428183; doi:10.1371/journal.pone.0236362)
Supplement: Attachment — Submitted filename: Response to reviewers.docx [file pone.0236362.s004.docx]

# Authors’ reply Academic Editor: Frederick Quinn PLOS ONE

# Dear Editor

We would like to thank the reviewers for their in-depth comments and suggestions. The comments have enabled us to improve the quality of the manuscript (PONE-D-20-06975R1). In this authors’ reply, we have tried as much as possible to give a point- by- point reply for each reviewer’s comments. The Reviewer’s comments are written in black-colored and the author’s replies are written in red-colored. The uploaded files include “revised manuscript with track changes” using red colored text showing all changes made (deleted sentences and phrases) and a clean version with filename “Manuscript” with no colored text have been uploaded.

6. Review Comments to the Author

Please use the space provided to explain your answers to the questions above. You may also include additional comments for the author, including concerns about dual publication, research ethics, or publication ethics. (Please upload your review as an attachment if it exceeds 20,000 characters)

Reviewer #1: I suggest to change the title to "Characteristics of multidrug-resistant tuberculosis patients in Tigray region, Ethiopia".

Comment accepted. The title is changed from “Multidrug-resistant Tuberculosis and Its Associated Factors from Tuberculosis Patients in Tigray Region, Ethiopia: a cross sectional study” to ". Characteristics of Multidrug-resistant Tuberculosis Isolates from Pulmonary Tuberculosis Patients in Tigray Region, Ethiopia: a cross-sectional study". We have added the term “Pulmonary Tuberculosis” because we did not include extra-pulmonary tuberculosis patients.
Reviewer #2: 1) If the lower risk of DR-TB with alcohol consumption is likely a statistical noise, as the authors acknowledged and which likely is the case, then this may not be highlighted in the abstract.
Comment accepted. We deleted alcohol consumption from the abstract.

2) In the discussion in lines 461-468, authors attribute the lack of association of sociodemographic factors to possible effect modification by geography. It is hard to conceive that the effect of age, residence, and education status on MDR TB would be different from region to region in Africa or Ethiopia. Looks like authors are using the term effect modification loosely without underlying reason or a basis. Authors can simply say they did not find association between sociodemographic factors and MDR TB prevalence. It is not necessary scientifically and biologically that MDR TB prevalence should be higher or lower in with increasing age; there are communities where actually MDR TB prevalence is higher in younger age group. There are many factors at play.

Comment accepted. We removed the reason why there is no association between sociodemographic factors and MDR-TB development in our finding.

3) Lines 466-468: "Besides, our finding is different from the global risk factors review report, and this variation among regionally and globally could be due to Effect modification by geographical area". Please pay attention to grammar.

Comment accepted. This sentence is already removed according to the above suggestion (2).
