# Supplementary material for: Characteristics of pulmonary multidrug-resistant tuberculosis patients in Tigray Region, Ethiopia: A cross-sectional study
Source: PLoS One. 2020 Aug 14;15(8):e0236362. doi: 10.1371/journal.pone.0236362 (PMC7428183; doi:10.1371/journal.pone.0236362)
Supplement: Attachment — Submitted filename: Response to Reviewers.docx [file pone.0236362.s005.docx]

# Authors’ reply Academic Editor: Frederick Quinn PLOS ONE

# Dear Editor

We would like to thank the reviewers for their in-depth comments and suggestions. The comments have enabled us to improve the quality of the manuscript (PONE-D-20-06975R2). In this authors’ reply, we have tried as much as possible to give a point- by- point reply for each reviewer’s comments. The Reviewer’s comments are written in black-colored and the author’s replies are written in red-colored. The uploaded files include “revised manuscript with track changes” using red colored text showing all changes made (deleted sentences) and a clean version with filename “Manuscript” with no colored text have been uploaded.

6. Review Comments to the Author

Please use the space provided to explain your answers to the questions above. You may also include additional comments for the author, including concerns about dual publication, research ethics, or publication ethics. (Please upload your review as an attachment if it exceeds 20,000 characters)

Reviewer #1: Regarding the title, I did not find that the authors described and discussed about charactersitics of isolate from MDRTB patient. If so, the manuscript should describe, discuss and explain more detail about microbiological aspects of the isolates. I read that the authors focused on the characteristics of the patients. Therefore, I suggest that the title should be revised to "Characteristics of pulmonary multidrug-resistant tuberculosis patients in Tigray Region, Ethiopia: a cross-sectional study". It will help the readers to understand the manuscript easily.

# Comment accepted. The title is changed from “Characteristics of multidrug-resistant tuberculosis isolates from pulmonary tuberculosis patients in Tigray Region, Ethiopia: a cross-sectional study” to "Characteristics of pulmonary multidrug-resistant tuberculosis patients in Tigray Region, Ethiopia: a cross-sectional study".
